# A Mechanistic Paradigm for Broad-Spectrum Antivirals that Target Virus-Cell Fusion

**DOI:** 10.1371/journal.ppat.1003297

**Published:** 2013-04-18

**Authors:** Frederic Vigant, Jihye Lee, Axel Hollmann, Lukas B. Tanner, Zeynep Akyol Ataman, Tatyana Yun, Guanghou Shui, Hector C. Aguilar, Dong Zhang, David Meriwether, Gleyder Roman-Sosa, Lindsey R. Robinson, Terry L. Juelich, Hubert Buczkowski, Sunwen Chou, Miguel A. R. B. Castanho, Mike C. Wolf, Jennifer K. Smith, Ashley Banyard, Margaret Kielian, Srinivasa Reddy, Markus R. Wenk, Matthias Selke, Nuno C. Santos, Alexander N. Freiberg, Michael E. Jung, Benhur Lee

**Affiliations:** 1 Department of Microbiology, Immunology and Molecular Genetics, University of California Los Angeles, Los Angeles, California, United States of America; 2 Department of Chemistry and Biochemistry, University of California Los Angeles, Los Angeles, California, United States of America; 3 Instituto de Medicina Molecular, Faculdade de Medicina da Universidade de Lisboa, Lisbon, Portugal; 4 Department of Biochemistry, Yong Loo Lin School of Medicine, National University of Singapore, Singapore; 5 NUS Graduate School for Integrative Sciences and Engineering (NGS), National University of Singapore, Singapore; 6 Department of Pathology, University of Texas Medical Branch, Galveston, Texas, United States of America; 7 Life Sciences Institute, National University of Singapore, Singapore; 8 Paul G. Allen School for Global Animal Health, Department of Veterinary Microbiology and Pathology, Washington State University, Pullman, Washington, United States of America; 9 Department of Chemistry and Biochemistry, California State University, Los Angeles, California, United States of America; 10 Department of Medicine, University of California Los Angeles, Los Angeles, California, United States of America; 11 Department of Cell Biology, Albert Einstein College of Medicine, Bronx, New York, United States of America; 12 Wildlife Zoonoses and Vector Borne Disease Research Group, Animal Health and Veterinary Laboratories Agency, Weybridge, Surrey, United Kingdom; 13 Oregon Health & Science University and VA Medical Center, Portland, Oregon, United States of America; 14 Department of Biological Sciences, Faculty of Science, National University of Singapore, Singapore; 15 Swiss Tropical and Public Health Institute and University of Basel, Basel, Switzerland; The Salk Institute for Biological Studies, United States of America

## Abstract

LJ001 is a lipophilic thiazolidine derivative that inhibits the entry of numerous enveloped viruses at non-cytotoxic concentrations (IC_50_≤0.5 µM), and was posited to exploit the physiological difference between static viral membranes and biogenic cellular membranes. We now report on the molecular mechanism that results in LJ001's specific inhibition of virus-cell fusion.

The antiviral activity of LJ001 was light-dependent, required the presence of molecular oxygen, and was reversed by singlet oxygen (^1^O_2_) quenchers, qualifying LJ001 as a type II photosensitizer. Unsaturated phospholipids were the main target modified by LJ001-generated ^1^O_2_. Hydroxylated fatty acid species were detected in model and viral membranes treated with LJ001, but not its inactive molecular analog, LJ025. ^1^O_2_-mediated allylic hydroxylation of unsaturated phospholipids leads to a *trans*-isomerization of the double bond and concurrent formation of a hydroxyl group in the middle of the hydrophobic lipid bilayer. LJ001-induced ^1^O_2_-mediated lipid oxidation negatively impacts on the biophysical properties of viral membranes (membrane curvature and fluidity) critical for productive virus-cell membrane fusion. LJ001 did not mediate any apparent damage on biogenic cellular membranes, likely due to multiple endogenous cytoprotection mechanisms against phospholipid hydroperoxides.

Based on our understanding of LJ001's mechanism of action, we designed a new class of membrane-intercalating photosensitizers to overcome LJ001's limitations for use as an *in vivo* antiviral agent. Structure activity relationship (SAR) studies led to a novel class of compounds (oxazolidine-2,4-dithiones) with (1) 100-fold improved *in vitro* potency (IC_50_<10 nM), (2) red-shifted absorption spectra (for better tissue penetration), (3) increased quantum yield (efficiency of ^1^O_2_ generation), and (4) 10–100-fold improved bioavailability. Candidate compounds in our new series moderately but significantly (p≤0.01) delayed the time to death in a murine lethal challenge model of Rift Valley Fever Virus (RVFV). The viral membrane may be a viable target for broad-spectrum antivirals that target virus-cell fusion.

## Author Summary

The threat of emerging and re-emerging viruses underscores the need to develop broad-spectrum antivirals. LJ001 is a non-cytotoxic, membrane-targeted, broad-spectrum antiviral previously reported to inhibit the entry of many lipid-enveloped viruses. Here, we delineate the molecular mechanism that underlies LJ001's antiviral activity. LJ001 generates singlet oxygen (^1^O_2_) in the membrane bilayer; ^1^O_2_-mediated lipid oxidation results in changes to the biophysical properties of the viral membrane that negatively impacts its ability to undergo virus-cell fusion. These changes are not apparent on LJ001-treated cellular membranes due to their repair by cellular lipid biosynthesis. Thus, we generated a new class of membrane-targeted broad-spectrum antivirals with improved photochemical, photophysical, and pharmacokinetic properties leading to encouraging *in vivo* efficacy against a lethal emerging pathogen. This study provides a mechanistic paradigm for the development of membrane-targeting broad-spectrum antivirals that target the biophysical process underlying virus-cell fusion and that exploit the difference between inert viral membranes and their biogenic cellular counterparts.

## Introduction

Advances in antiviral therapeutics have allowed for effective management of specific viral infections, most notably human immunodeficiency virus (HIV) [1]. Yet, the one-bug-one-drug paradigm of drug discovery is insufficient to meet the looming threat of emerging and re-emerging viral pathogens that endangers global human and livestock health. This underscores the need for broad-spectrum antivirals that act on multiple viruses based on some commonality in their viral life cycle, rather than on specific viral proteins. Recently, a few broad-spectrum antivirals have been described that target enveloped virus entry [2], [3], [4], [5], [6] or RNA virus replication [7], [8], [9], [10]. The former targets the viral membrane, or more precisely, the biophysical constraints of the virus-cell membrane fusion process, while the latter targets nucleic acid metabolic pathways.

LJ001 is a membrane-binding compound with broad-spectrum antiviral activity *in vitro*. LJ001 acts on the virus, and not the cell, inhibiting enveloped virus infection at the level of entry [4]. LJ001 is non-cytotoxic at antiviral concentrations, yet had the remarkable property of inhibiting all enveloped viruses tested, including those of global biomedical and biosecurity importance such as HIV, hepatitis C virus (HCV), Influenza, Ebola, henipaviruses, bunyaviruses, arenaviruses and poxviruses. LJ001 is also clearly not virolytic and does not act as a “detergent”: LJ001-treated virions remain intact and their viral envelopes functional, as LJ001-treated virions are still able to bind to their receptors. A panoply of assays showed that even though LJ001 was lipophilic, and could bind to both viral and cellular membranes, it inhibited virus-cell but not cell-cell fusion. This puzzling dichotomy was illuminated when studies with lipid biosynthesis inhibitors indicated that LJ001 was indeed cytotoxic when the ability of a cell to repair and turnover its membranes is compromised. Thus, we posited that the antiviral activity of LJ001 relies on exploiting the physiological difference between inert viral membranes and biogenic cellular membranes with reparative capabilities [4].

However, the molecular target of LJ001 remains to be defined, and a precise molecular mechanism that could explain the extraordinary breadth of LJ001's antiviral activity against lipid-enveloped viruses is lacking. This has limited consideration of the viral membrane as a plausible target for the development of broad-spectrum antivirals. Here, we identify the molecular target of LJ001 and present a strong body of evidence that supports a unifying hypothesis regarding its mechanism of action. Based on this mechanistic understanding, structure-activity relationship (SAR) optimization resulted in a new class of membrane-targeted broad-spectrum antivirals with markedly enhanced potencies and other relevant biophysical and pharmacokinetic properties that underscore the veracity of our mechanism of action (MOA) hypothesis. Finally, we validated our hypothesis *in vivo* by interrogating the efficacy of this new class of membrane-targeted antivirals against a virulent (enveloped) viral pathogen in a lethal challenge animal model.

## Results

### LJ001 inhibits a late stage of viral fusion

**Figure 1:**
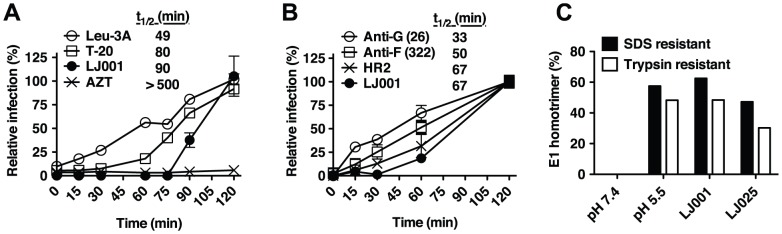
LJ001 inhibits a late stage of viral fusion. (**A**) Time-of-addition experiment (see Figure S1). HIV-1_JRCSF_ infection of TZM-bl cells was synchronized by spinoculation for 2 h at 4°C. The plates were subsequently incubated at room temperature (t = 0) for the first 60 min, then to 37°C. LJ001 (20 µM) or HIV entry inhibitors specifically blocking CD4-attachment (Leu-3A, 10 µg/ml), or 6-HB formation (T-20 or enfuvirtide, 5 µM) were added at different times. AZT (10 µM) blocks reverse transcription, a post-entry step. Luciferase expression in cell lysates 48 h post-infection was expressed relative to untreated control (100%). Data representing the mean ± SD of triplicate experiments were graphed, and *t*_1/2_ values calculated using GraphPad PRISM. (**B**) VSV-ΔG-rluc pseudotyped with NiV envelope glycoproteins, F and G, was spinoculated for 2 h at 4°C onto VERO cells to synchronize the infection. The plates were subsequently shifted to room temperature (t = 0) for 1 h before incubating at 37°C. Inhibitors of NiV entry specifically blocking: attachment (Anti-G, Mab26, 1 µg/ml), fusion triggering (Anti-F, Mab322, 1 µg/ml), or 6-HB formation (HR2, peptide equivalent of T-20 in the HIV system, 1 µM) [12], [51], and LJ001 (10 µM) were added at different times. Luciferase expression in cell lysates was analyzed 24 h post-infection and expressed relative to untreated control (100%). Data representing the mean ± SD of duplicate experiments were graphed, and *t*_1/2_ values calculated using GraphPad PRISM. (**C**) Radiolabeled SFV treated with 6.15 µM of LJ001, or the inactive control LJ025, was allowed to adsorb to BHK cells on ice. After washing, membrane fusion was triggered by low pH, 1 min at 37°C. Controls included non-treated cell-bound virus incubated at low or neutral pH. After fusion triggering, cell lysates were collected and the trypsin- and SDS-resistant E1 homotrimer in each sample was quantified by SDS-PAGE and phosphorimaging. Results, representative of two independent experiments, are expressed as a percent of the total E1 present.

To further define the molecular mechanism of LJ001's antiviral activity, we first investigated where LJ001 acts during the fusion cascade. A time-of-addition experiment, schematically shown in Figure S1, indicated that LJ001 inhibited the HIV fusion cascade at a step subsequent to CD4-receptor binding and pre-hairpin intermediate (PHI) formation (Figure 1A). Thus, the inhibitory half-life of LJ001 was longer than that of a CD4 blocking antibody (Leu3A) and T-20, a heptad-repeat (HR)-derived peptide that targets the PHI and prevents six-helix bundle formation (6-HB) [11]. LJ001 similarly inhibited Nipah virus (another Class I fusion protein) envelope mediated entry [12], although in this case, the resolution of our assay couldn't distinguish between PHI and 6-HB formation (Figure 1B). These results suggest LJ001 acts late in the fusion cascade, likely after PHI formation. LJ001 also acts late in the Class II fusion protein cascade, as we found that it did not affect homotrimer formation of the Semliki forest virus (SFV) E1 protein (Figure 1C), even at concentrations that completely inhibited virus fusion (Figure S2). Class II E1 homotrimer formation is analogous to six-helix bundle (6-HB) formation for Class I fusion proteins and marks a late step in the fusion cascade [13], [14]. These data confirm that LJ001 inhibits both Class I and II fusion, highlight that LJ001 abrogates viral infectivity while maintaining the conformational integrity of the viral envelopes, and demonstrate that LJ001 inhibits fusion at a very late stage, likely just prior to virus-cell membrane merger.

### LJ001 oxidizes unsaturated fatty acids in viral membranes

**Figure 2:**
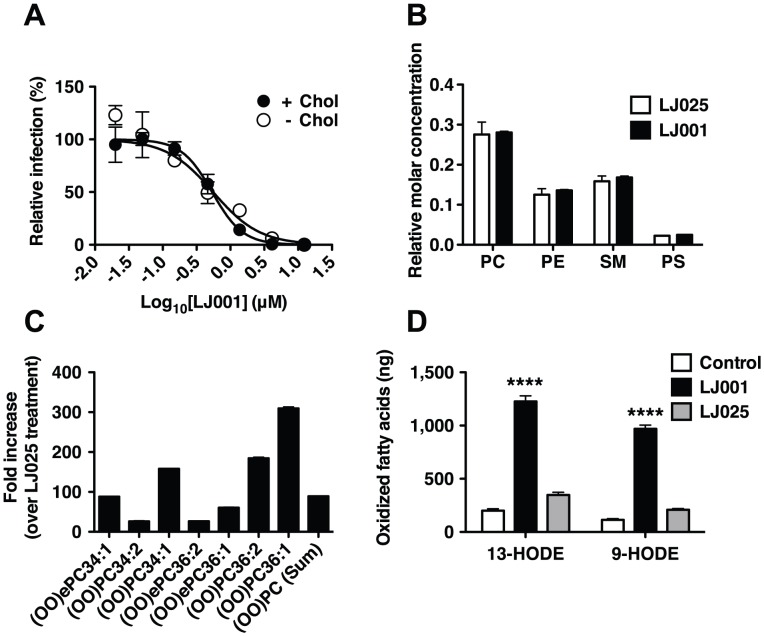
LJ001 oxidizes unsaturated fatty acids in viral membranes. (**A**) Equivalent titers of Semliki forest virus (SFV) grown in cholesterol-depleted or control C6/36 mosquito cells were treated with increasing concentrations of LJ001 and their infectivity on target BHK cells determined by immunofluorescence (as in Figure S2). Results are presented as % of infection (mean ± SD, n = 3) relative to that obtained in the absence of LJ001 treatment. The IC_50_ for LJ001's antiviral activity was determined by non-linear regression using GraphPad PRISM (Top = 100%, Bottom = 0%). (**B–C**) Purified influenza A virus (A/PR/8/34 H1N1) was treated with 5 µM of LJ001, or control LJ025, and exposed to light for 1 h. The total lipid content was extracted and the viral lipidome analyzed by high-resolution LC-MS (see Figure S3). (**B**) Relative molar concentration of the major phospholipid species present in the viral lipidome. (**C**) The amount of peroxidized phosphatidylcholine (PC) species, presented as fold-increase in LJ001- over LJ025-treated samples. Similar results were obtained in two independent experiments with two technical replicates each. PE: Phosphatidylethanolamine, PS: Phosphatidylserine, SM: Sphingomyelin, (OO)ePC: oxidized (hydroperoxide) ether PC, (OO)PC oxidized (hydroperoxide) PC. (**D**) Liposomes (150 µg in 1 ml) were treated with LJ001 (10 µM), or control LJ025, and exposed to light for 1 h. After de-esterification, fatty acids were extracted, and the amount of 9-hydroxy-10E,12Z-octadecadienoic acid (9-HODE) and 13-hydroxy-9Z,11E-octadecadienoic acid (13-HODE) was determined by LC-MS/MS. Data represents the mean ± SD of triplicates. ****: p<0.0001, LJ001 vs LJ025, Two-way ANOVA, Bonferroni post-test using GraphPad PRISM.

Lipid composition can affect the biophysical properties of viral membranes that impact the efficiency of virus-cell fusion. Insect cells are cholesterol auxotrophs and can be grown in the absence of sterols, and thus, SFV can be generated with or without cholesterol in viral membranes. The sensitivity of SFV to LJ001 did not differ significantly between viruses grown in the presence or absence of cholesterol (Figure 2A), suggesting that cholesterol is not a membrane component essential for LJ001's antiviral activity. To determine if LJ001 affected the phospholipid composition of viral membranes, we treated influenza virus A (A/PR/8/34 H1N1) with LJ001 or its inactive analog, LJ025 [4], and analyzed the viral lipidome by mass spectrometry after liquid chromatography separation (LC-MS). No difference was observed in the overall phospholipid composition of treated viruses (Figure 2B). However, high-resolution LC-MS spectral analysis revealed that LJ001-treated viruses had up to 300-fold increase in the number of *oxidized* forms of unsaturated phospholipids, compared to LJ025-treated samples (Figure 2C and Figure S3). To rule out other virus-specific or virion-associated co-factors, we used liposomes with a defined phospholipid composition, and showed that LJ001 could mediate the specific and direct oxidation of linoleic acid (18∶2) (Figure 2D), an unsaturated fatty acid present in viral and cellular membranes [15], [16], [17].

### The antiviral activity of LJ001 is dependent on its ability to generate singlet oxygen

**Figure 3:**
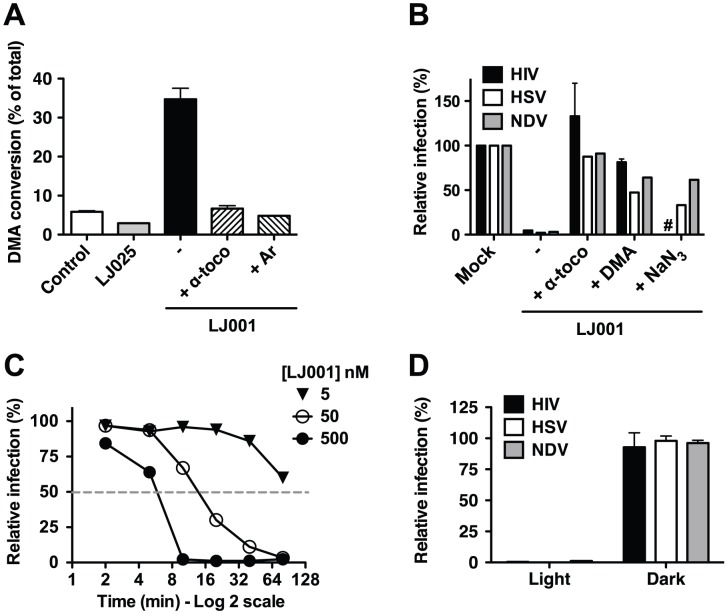
The antiviral activity of LJ001 is dependent on its ability to generate singlet oxygen (^1^O_2_). (**A**) LJ001, or control LJ025, was added to a solution of DMA and kept under light. After 6 h, DMA conversion was detected by ^1^H-NMR (DMA∶oxiDMA = 3.1 ppm:2.1 ppm (methyl peak)). Reactions were performed in CDCl_3_ using 1 equivalent of each reagent. CDCl_3_ was saturated with oxygen by bubbling O_2_ through the solvent for 30 min and the reaction was kept under O_2_ gas atmosphere, except for Ar where oxygen was exchanged with argon by freeze/thaw method. Data represents the mean ± SD of duplicate experiments. (**B**) HIV-1_IIIB_, Herpes Simplex Virus-1 (HSV) or Newcastle disease virus (NDV) were incubated with 0.25 µM of LJ001 in the presence of 50 µM α-tocopherol or DMA, or 100 mM NaN_3_. Infectivity was determined as described in Materials and Methods, and results presented as infection relative to untreated virus (100%). HIV: mean ± SD of duplicate measurements, representative of three independent experiments. HSV and NDV: results representative of three independent experiments. #: NaN_3_ was toxic to TZM-Bl cells used to assay HIV entry. (**C**) HSV was incubated with 5, 50 or 500 nM of LJ001 and exposed to white light for 2, 5, 10, 20, 40 or 80 min. Infectivity was determined as described in Materials and Methods, and results presented as infection relative to untreated virus (100%) at a given time, to account for loss of infectivity over time, and as a function of time of light exposure. Data are representative of two independent experiments. (**D**) HIV-1_IIIB_, HSV or NDV were treated in the dark with 1 µM of LJ001, and subsequently either exposed to a white light source or left in the dark, for 10 min, before infection of cells in the dark. Relative infectivity was determined as in (**B**). LJ001-treated viruses exposed to light had >99% reduction in infectivity. Data represents the mean ± SD of two independent experiments.

Reactive oxygen species such as singlet oxygen (^1^O_2_) are known to react readily with carbon-carbon double bonds (alkenes) present in the acyl chains of unsaturated phospholipids, and this process would generate the oxidized phospholipids described in Figures 2C–D. To evaluate the capacity of LJ001 to generate ^1^O_2_, we added LJ001 to 9,10-dimethylanthracene (DMA), a specific ^1^O_2_ trap, and quantified the oxidation of DMA by ^1^H-NMR (Figure 3A and Figure S4). LJ001, but not LJ025, exhibited ^1^O_2_-mediated oxidation of DMA, which was decreased by the antioxidant α-tocopherol (α-toco) and absent when molecular oxygen was replaced by argon (Ar). Correspondingly, the ability of LJ001 to inhibit multiple viruses was abrogated not only by the addition of a lipophilic antioxidant (α-toco) or ^1^O_2_ quencher (DMA), but also by a water-soluble ^1^O_2_ quencher (NaN_3_) (Figure 3B). Thus, we hypothesized that LJ001's antiviral activity is attributable to its properties as a type II photosensitizer [18], [19], a compound that generates highly reactive excited-state ^1^O_2_ by transferring energy of the excited sensitizer to ground-state (triplet) molecular oxygen (^3^O_2_). Our hypothesis predicts that as a photosensitizer, LJ001's antiviral activity should also be dependent on light. Indeed, the antiviral activity of LJ001 was dependent on both its concentration and the time-of-exposure to white light. For example, doubling the time of light exposure achieved the same viral inhibitory effect at ten-fold lower concentrations (Figure 3C, compare 50 and 500 nM curves). Importantly, LJ001's antiviral activity was absent when no visible light source was used (Figure 3D). Since LJ001 membrane intercalation is dictated by its lipophilic properties and not the presence of light, this latter observation underscores our previous observations [4] that, at the active concentrations used, membrane insertion itself does not account for the antiviral activity of LJ001. Finally, to provide independent confirmation of the type II photosensitizing properties of LJ001, we subjected a solution of LJ001 in CD_2_Cl_2_ under ambient conditions to flash excitation, and observed the characteristic ^1^O_2_ emission in the near-infrared (Figure S5).

### The effect of LJ001 on the biophysical properties of model versus cellular membranes

**Figure 4:**
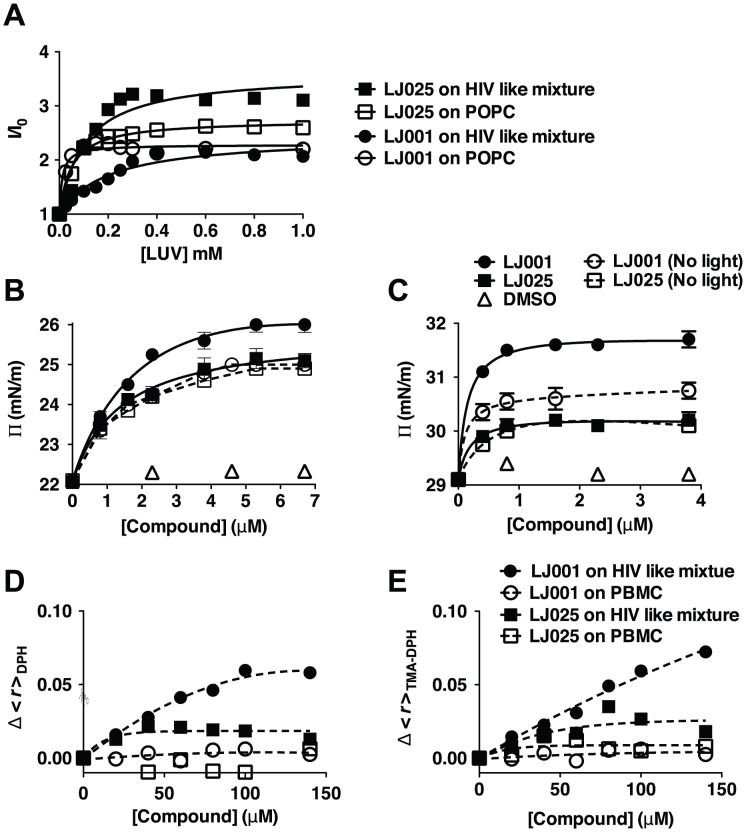
The effect of LJ001 on the biophysical properties of model versus cellular membranes. (**A**) Relative fluorescence intensity increase, of the sample compounds in the presence (*I*) or absence (*I_0_*) of the indicated amounts of membrane, due to partition of LJ001 and LJ025 into large unilamellar vesicles (LUV), performed by successive additions of a concentrated LUV suspension of pure POPC (1-palmitoyl-2-oleyl-*sn*-glycero-3-phosphocholine, a lipid with packing density and fluidity properties similar to mammalian cell membranes) or HIV membrane-like mixture (POPC 5.3%, DPPC 3.5%, cholesterol 45.3%, SM 18.2%, POPE 19.3% and POPS 8.4%; mol %[15]). Data are representative of three independent experiments. The partition coefficients (K_p_) and the fluorescence intensity ratios (I_Lipids_/I_Water_) resulting from the curve fitting shown here can be found in Table S1. (**B–C**) Surface pressure measurements on a lipid monolayer comprised of (**B**) pure POPC or (**C**) HIV membrane-like mixture with increasing addition of LJ001, LJ025, or DMSO (vehicle control), in the presence or absence of light. Data represent the mean ± SD of duplicate measurements and are representative of three independent experiments. (**D–E**) Changes in fluorescence anisotropy (<*r*>) as a function of LJ001 or LJ025 addition to LUV with HIV membrane-like mixture or peripheral blood mononuclear cells (PMBC) using the fluorescent probes (**D**) DPH or (**E**) TMA-DPH. Control measurements of <*r*> vs temperature, using LUV of a reference lipid, showed that the probes were able to correctly detect the membrane phase transition, demonstrating that the compounds did not interfere with the correct assessment of membrane fluidity. Each point is the average of at least triplicates of independent samples.

We propose that after insertion into the viral membrane, light activation of LJ001 triggers the generation of ^1^O_2_ that oxidizes the unsaturated chains of fatty acids composing the phospholipids of the viral membrane. In further support of our model, we showed that LJ001 (and LJ025) efficiently partitions into model lipid membranes mimicking the lipid packing density, fluidity, and composition of viral (HIV-like) or cell (POPC) membranes (Figure 4A and Table S1). Indeed, when lipid membranes were non-limiting (>50-fold molar excess of lipid), over 85% of LJ001 or LJ025 were protected from the water-soluble quencher (acrylamide), and thus, completely buried in the lipid bilayer (Figure S6). ^1^O_2_-mediated oxidation of unsaturated phospholipids proceeds by a “singlet oxygen ene” reaction, resulting in a *cis*-to-*trans* isomerization of a double bond in the unsaturated fatty acids and the presence of a polar group (hydroperoxy- or hydroxy-) in the hydrophobic core of the lipid bilayer (Figure S7, first and second panel). *Cis*-to-*trans* isomerization allows for closer packing of the fatty acid acyl chains in the lipid bilayer, which could result in a tighter positive curvature, while lipid oxidation results in clustering of the oxidized lipids into microdomains, reducing exposure of the polar groups to the hydrophobic acyl chains in the lipid bilayer core (Figure S7, third and fourth panel) [20]. The latter effectively reduces membrane average fluidity (and/or increases rigidity), as lipid species are now not as freely diffusible. Indeed, surface pressure and steady-state fluorescence anisotropy measurements indicated that LJ001 induced tighter lipid packing (Figure 4B–C), and reduced membrane fluidity (Figure 4D–E) of various model lipid monolayers, significantly more than LJ025. These effects were especially prominent using HIV membrane-like mixtures. Importantly, LJ001 did not show an effect on lipid packing when not exposed to light (“dark” in Figure 4B–C), and neither compounds affected membrane fluidity when tested on biogenic cellular membranes (primary peripheral blood mononuclear cells (PBMC) obtained from blood donors, Figure 4D–E). The former confirms that membrane insertion alone does not account for the change in membrane biophysical properties mediated by LJ001, and the latter is consistent with our prior observations [4] that LJ001 damages inert viral membranes but not biogenic cellular membranes. In light of our elucidation that LJ001 acts as a lipophilic photosensitizer, the explanatory mechanism becomes clear: cells have multiple endogenous cytoprotection mechanisms against phospholipid hydroperoxides [21] that can overcome the oxidative damages done by LJ001 to cellular membrane lipids, whereas viral membranes have no such reparative capacity to guard against LJ001-mediated oxidative damage. *In toto*, these data indicate that LJ001 is a light-activated membrane-intercalating photosensitizer that catalyzes ^1^O_2_-mediated lipid oxidation of unsaturated phospholipids; this results in changes to the biophysical properties of the viral membrane that negatively impacts its ability to undergo virus-cell fusion.

### Improving the antiviral and photophysical properties of membrane-targeted photosensitizers

**Figure 5:**
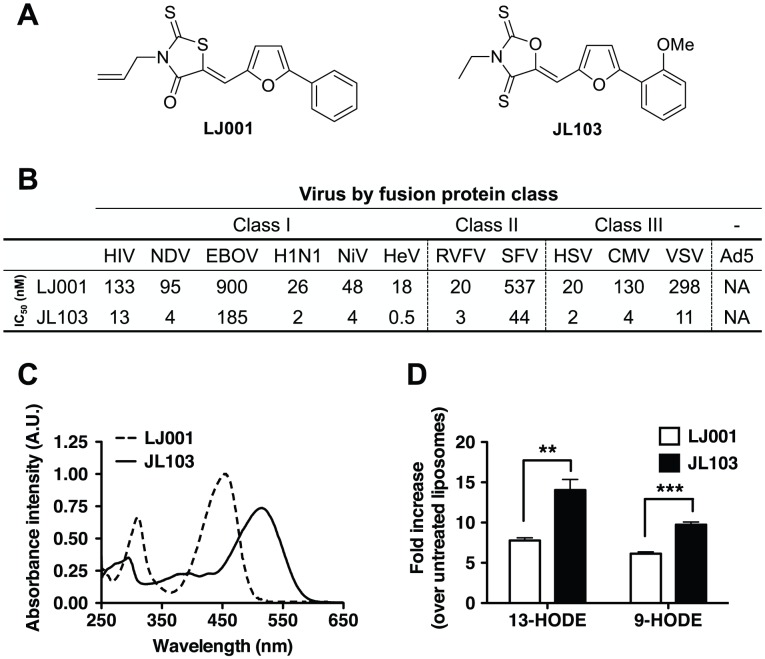
Improved antiviral and photophysical properties of the oxazolidine-2,4-dithione JL103. (**A**) Structures of LJ001 and JL103. (**B**) IC_50_ of LJ001 and JL103 against representative viruses that use different classes of fusion proteins (see Figure S8). (**C**) Absorption spectra of LJ001 and JL103 (100 µM in DMSO). (**D**) Liposomes (150 µg in 1 ml) were treated with JL103 or LJ001 (10 µM) and exposed to light for 1 h. Fatty acids were extracted as in Figure 2D, and the amount of 9- and 13-HODE was determined by LC-MS/MS. Results are shown as the fold-increase (mean ± S.D., n = 3) in oxidized lipids over untreated samples. Student's t test: **, p = 0.0097; ***, p = 0.0009.

Having established that the broad-spectrum antiviral activity of LJ001 was due to its properties as a membrane-targeted photosensitizer, we sought to increase its antiviral potency by structure-activity relationship (SAR) experiments. LJ001 is a rhodanine derivative; rhodanines are derivatives of thiazolidines, such as the 5-membered ring on the left hand side of LJ001 (Figure 5A). In order to maximize the absorption, and perhaps also shift the peak absorption (λ_max_) to longer tissue-penetrating wavelengths, we decided to investigate other ring systems analogous to the thiazolidine unit of the rhodanines. In particular we wanted to change the sulfur atom in the ring to a smaller atom, e.g., nitrogen or oxygen to perhaps have better electronic overlap. While the imidazolidine (nitrogen in the ring) analogues had essentially no activity (data not shown), we found that the oxazolidine analogues (oxygen in the ring) had superior activity. We therefore carried out a small SAR study of the 5-(5-arylfurfurylidene)-2-thioxooxazolidin-4-one and the analogous 5-(5-arylfurfurylidene)oxazolidine-2,4-dithiones (see Text S1) that led us to an *oxa*zolidine-2,4-dithione we named **JL**103 (Figure 5A). Although it was still inactive against a non-enveloped virus (Adenovirus serotype 5, Ad5), JL103 maintained the broad-spectrum activity of LJ001 against enveloped viruses—from all three classes of fusion proteins—with at least a 10-fold increase in potency (Figure 5B and Figure S8). JL103 was also mechanistically similar to LJ001 (Figure S9 and Tables S1 and S2): (i) it remained a membrane-targeted photosensitizer and its antiviral activity still required the presence of light, (ii) its antiviral activity could be reduced by antioxidants, and (iii) it acted on a similarly late stage of the HIV fusion cascade, but likely with a better efficiency than LJ001 at the same concentration. However, we noted a few differences that were mechanistically illuminative: the addition of a somewhat polar but uncharged substituent (methoxy) to the right-hand phenyl ring in JL103 decreased its partitioning into membranes (Table S1); nevertheless, JL103's ability to generate ^1^O_2_ at a higher rate than LJ001 (Figure S9 and Table S2) indicates that this increased quantum yield is the dominant factor that contributes to the enhanced antiviral potency of JL103.

Analysis of JL103's photophysical properties indicated that its absorption spectrum was red-shifted (Figure 5C; λ_max_, LJ001 = 455 nm, JL103 = 515 nm), and that the total integrated absorption (AUC) within the optical spectrum (λ = 400 to 750 nm) was 1.53 times that of LJ001 (Table S2). Flash excitation of a solution of JL103 in CD_2_Cl_2_ under ambient conditions also resulted in the characteristic ^1^O_2_ emission in the near-infrared (data not shown), confirming that JL103 is a *bona fide* ^1^O_2_ generator. However, compared to LJ001, JL103 had improved ^1^O_2_ quantum yields (QY) at both 355 and 532 nm (Table S2). These results confirm that JL103 is more efficient in generating ^1^O_2_ than LJ001 [18], [19]. Consequently, under the same conditions, JL103-treated liposomes had significantly more oxidized lipids than LJ001-treated liposomes (Figure 5D), implicating the enhanced photosensitizing properties of JL103 in its increased antiviral potency. Of note, these photosensitizers have relatively small rates of ^1^O_2_ removal (*k*_T_, Table S2) indicating that self-quenching of ^1^O_2_ by the photosensitizer-drug was not significantly limiting their antiviral function.

### Overcoming the hemoglobin barrier for the *in vivo* use of membrane-targeted photosensitizers as antivirals

**Figure 6:**
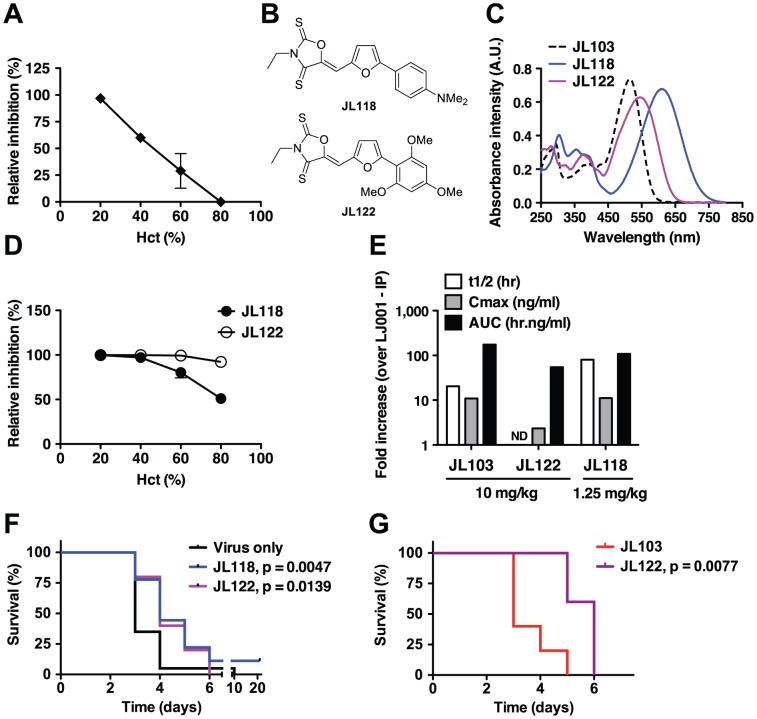
Evaluation of candidate oxazolidine-2,4-dithiones for antiviral activity *in vivo*. (**A**, **D**) Antiviral efficacy of (**A**) JL103 or (**D**) JL118 and JL122 at varying hematocrits (Hct). RBCs in PBS were spiked with HIV-1_JR-CSF_ and brought to the indicated Hct. Normal human Hct is 45±5%. Thin layers of spiked RBCs were treated with 20 µM of the indicated compound under light, for 1 h under constant agitation. Remaining infectivity in the supernatant of treated RBCs was evaluated by inoculating reporter TZM-bl cells. Data represent the relative infectivity (mean ± SD, n = 2) measured 48 h post-infection (untreated control = 100%) from one of two representative experiments. (**B**) Structures of JL118 and JL122. (**C**) Absorption spectra of JL103, JL118 and JL122 (100 µM in DMSO). (**E**) Pharmacokinetics of JL103, JL122 and JL118 in mice. ND: Not determined. (**F**) Mice lethally challenged IP with 20 pfu of RVFV ZH501 were treated IP once a day for 7 days, starting 1 h post-challenge, with JL118 (1.25 mg/kg) or JL122 (10 mg/kg). n = 20 per group. (**G**) Mice lethally challenged IP with 50 pfu of RVFV ZH501 were treated IP at 1, 12, 24 and 48 h post-challenge with JL103 (10 mg/kg) or JL122 (10 mg/kg). n = 5 per group. For both (**F**) and (**G**), mice were monitored daily and survival as a Kaplan-Meier plot was compared with the Log-rank (Mantel-Cox) test using GraphPad PRISM. Respective p values are indicated on the graphs. (**F**) JL118 or JL122 treatment moderately, but significantly, increased median survival times compared to the untreated group. (**G**) Median survival significantly increased from 3 to 6 days for JL103- vs JL122-treated mice, respectively.

Oxazolidine-2,4-dithiones (*e.g.* JL103) are novel non-rhodanine compounds that are more potent inhibitors of virus-cell fusion than the rhodanine derivatives (*e.g.* LJ001) we previously characterized as broad-spectrum antivirals [4]. Despite the increased potency and enhanced photosensitizing properties of JL103, we thought it unlikely that JL103 (λ_max_ = 515 nm) would exhibit antiviral activity neither *in vivo* nor in the common use of photosensitizers for whole blood or packed red blood cells (RBC) decontamination, known as Pathogen Reduction Technology (PRT), as the hemoglobin present in molar excess would compete effectively for any incident photons with wavelengths <600 nm [19]. To confirm the competitive effect of hemoglobin, we tested the antiviral efficacy of JL103 in the presence of increasing amounts of human RBC. Indeed, the antiviral efficacy of JL103 was inversely proportional to the hematocrit (Hct), and at physiological Hct (∼45% RBC v/v), the antiviral activity of JL103 was reduced by >50% (Figure 6A). To rule out that this reduction in antiviral activity was not simply due to competition by the increasing amount of RBC membranes, we performed a second SAR study with the aim of developing new oxazolidine-2,4-dithiones with even more red-shifted absorption spectra. We hypothesized that compounds with equivalent ^1^O_2_ quantum yields, but with absorption spectra that extend beyond ∼600 nm, would maintain the potency of JL103 even at physiological hematocrits.

The structures of the new JL compounds (oxazolidine-2,4-dithiones) are given in Figure S10 and their antiviral activity (IC_50_), cytotoxicity to primary PBMCs (CC_50_), and therapeutic indexes (TI) in Table S3. We generated a series of active oxazolidine-2,4-dithiones by modulating the electron-donating nature of the substituents on the right-hand phenyl ring. Thus, JL108 (4-methoxy), JL109 (2,4-dimethoxy), JL122 (2,4,6-trimethoxy), and JL118 (4-dimethylamino) were all as potent as JL103, if not more, when tested against a representative panel of enveloped viruses (Table S3). Interestingly, these compounds exhibited increasingly red-shifted absorption spectra with λ_max_ ranging from 530 (JL108) to 550 (JL109), 545 (JL122), and 610 (JL118) nm (Figure S11 and Table S2) (note: λ_max_ for LJ001 and JL103 is 455 and 515 nm, respectively). All these compounds were also confirmed to be ^1^O_2_ generators with equivalent or greater quantum yields when compared to JL103 (Table S2). We chose to follow-up on JL118 and JL122 (Figure 6B) as they represent different classes of phenyl substituents (dimethylamino versus methoxy), and were both at least as potent as JL103 in their antiviral activity, but had red-shifted absorption spectra beyond those of JL103 and hemoglobin (Figure 6C). Indeed, in contrast to JL103, and consistent with our hypothesis, JL118 and JL122 maintained their antiviral potency at physiological hematocrits (Figure 6D). These results provide independent confirmation that the negative correlation seen in Figure 6A, between the antiviral activity of JL103 and Hct, was *not* simply due to the presence of extra RBC membranes, but indeed resulted from the hemoglobin competing for incident photons. JL118 and JL122 still insert into membranes, as indicated by their partitioning into membranes (Table S1), with K_p_ values between those of LJ001 and JL103.

### Evaluating the *in vivo* efficacy of candidate oxazolidine-2,4-dithiones

As the addition of somewhat polar but uncharged substituents (methoxy or dimethylamino) to the phenyl ring may also improve the solubility and bioavailability of the compounds, we evaluated the pharmacokinetics of candidate compounds. Indeed, JL103, JL118 and JL122 all exhibited >10-fold improvements in relevant pharmacokinetic (PK) parameters compared to LJ001 (longer half-life, better AUC, improved bioavailability and lower clearance, see Figure 6E and Table S4). Thus, we evaluated their potential antiviral activity in a stringent lethal challenge model of Rift valley fever virus (RVFV), where the median lethal dose (LD_50_) was ≤1 pfu (plaque forming unit) (Figure S12). In mice lethally challenged with 20×LD_50_ of RVFV, treatment with JL118 or JL122 resulted in a moderate but significant delay in time-to-death compared to untreated controls (Figure 6F). As expected, treatment with JL103 had no significant effect on survival (Figure S12), indicating that the absorption spectrum of the compound plays a critical role in its antiviral activity *in vivo*. Furthermore, even at a higher challenge dose (50×LD_50_), JL122 treatment still resulted in a significant delay in time-to-death when compared to JL103 treatment (Figure 6G), suggesting that the red-shifted absorption spectra of JL122 and JL118 likely accounts for their improved antiviral activity *in vivo* compared to JL103. Recall that JL103, JL118 and JL122 all had similar PKs and *in vitro* IC_50_ values against diverse species of enveloped viruses (Figure 6E and Tables S3 and S4).

## Discussion

LJ001 was previously reported to be a small molecule broad-spectrum antiviral that targets entry of lipid-enveloped viruses [4]. Despite careful characterization of LJ001's antiviral properties, the molecular target and mechanistic basis for the broad-spectrum activity of LJ001 remained elusive.

Here, we identify the unsaturated fatty acid chains of viral membrane phospholipids as the major targets of LJ001's antiviral activity. Furthermore, we not only confirmed that LJ001 insertion into membranes is necessary but not sufficient for its antiviral activity [4], but also provided evidence for a unifying mechanistic hypothesis that accounts for the broad-spectrum antiviral activity of LJ001 against enveloped viruses. LJ001 acts as a membrane-targeted photosensitizer: the phospholipid modifications, resulting from the light-dependent LJ001-induced ^1^O_2_-mediated lipid oxidation, negatively impact on the fine-tuned biophysical properties of viral membranes critical for productive virus-cell membrane fusion (e.g. by increasing membrane curvature and/or decreasing fluidity). Thus, the photosensitizing properties of LJ001 mediate its antiviral activity. Our proposed mechanism of action provides an explanatory basis for our observation that while LJ001 can clearly bind to both cellular and viral membranes, it is not cytotoxic to cells at antiviral concentrations unless the ability of the cell to repair its membranes is compromised [4]. This mechanism is consistent with our model that LJ001's antiviral activity exploits the inability of static viral membranes to repair LJ001-mediated damage, and also explains why this class of broad-spectrum antivirals affects virus-cell, but not cell-cell fusion [4]. Indeed, the effects of oxidized phospholipids on the biophysical properties of membranes (Figure 4) are only apparent on viral membranes, and not on biogenic cellular membranes (*e.g.* PBMCs), which are subject to repair, turnover, and translocation processes. These latter mechanisms have evolved to mitigate the negative effects posed by oxidized phospholipids [21].

Our mechanistic model for LJ001's mode of action was further confirmed by SAR experiments. We developed a new class of membrane-targeted broad-spectrum antivirals where, as hypothesized, the enhanced antiviral activity was correlated with improved ^1^O_2_ quantum yields, and more favorable photochemical and photophysical properties. These improvements overcame some of the limiting barriers that previously restricted the *in vivo* antiviral efficacy of this class of photosensitizers. Indeed, in proof-of-principle studies, we showed that JL118 and JL122, from the new JL-series of membrane-targeted photosensitizing compounds, not only were more effective at inactivating HIV in the presence of a large excess of RBC (i.e. hemoglobin), but also moderately, yet significantly, prolonged the time-to-death in a lethal challenge model of RVFV. Importantly, the demonstrated *ex vivo* and *in vivo* antiviral efficacy of JL118 and JL122 compared to JL103 provides functional validation of our SAR strategy, and is consistent with the panoply of *in vitro* assays that supports our model for the molecular mechanism that underlies the broad-spectrum antiviral activity of our novel series of membrane-targeted photosensitizers.

Photosensitizers have been used clinically in many forms of photodynamic therapy. The majority of photosensitizers in clinical use focus on their ability to damage nucleic acids or proteins. There is also a large literature on membrane-targeted photosensitizers; many of them are porphyrin derivatives. Benzoporphyrin derivative monoacid ring A (BPD-MA) is a photosensitizer that has long been known to be a virucidal agent *in vitro* [22]. Remarkably, verteporfin, another BPD, was recently evaluated as an agent in extracorporeal photopheresis in HIV-infected patients, and shown to have a significant impact on viral load in a subset of patients that underwent an extended treatment course [23], [24]. Due to logistical and practical considerations, photodynamic therapy to reduce viral pathogen load is unlikely to be an efficient application for chronic infections like HIV. However, our JL compounds with absorption spectra that are red-shifted beyond that of hemoglobin may warrant further evaluation of their use in PRT for transfusion medicine [25]. For example, whereas testing and PRT for blood products using photosensitizers are common in developed countries, they remain, as currently constituted, expensive and unaffordable in resource-poor countries, where blood-borne pathogens transmissions during transfusions is still present at unacceptable rates. Thus, the identification, development and testing of more affordable photosensitizers that can sustain greater variability in quality control processes are highly desirable. Incidentally, our experiments showing that JL118 and JL122 still maintained effective antiviral activity even at high hematocrits, and in the presence of just white ambient light, may provide proof-of-principle of this application.

To our knowledge, despite the large literature on membrane-targeted photosensitizers and many claims as to their use as virucidal agents, no one has precisely identified the molecular mechanisms by which specific membrane-targeted photosensitizers inhibit virus-cell fusion [26]. In addition, the putative anti-viral activity of photosensitizers such as Hypericin and Rose Bengal, Hypocrellin A, Methylene Blue derivatives or Phthalocyanines, to name a few, has always been examined at concentrations at least 2 logs higher than what we have used for JL118 and JL122, and their antiviral activity generally attributed to singlet oxygen's, or other ROS', effects on proteins and/or nucleic acids [27], [28], [29], [30], [31]. Herein, we elucidated the molecular and biophysical mechanisms that underlie the antiviral activity of a well-known class of compounds: membrane-intercalating photosensitizers. In so doing, we generated a novel class of such compounds (**oxazolidine-2,4-dithione** derivatives) with effective nM IC_50_s, and showed that improving the relevant photophysical and photochemical properties can lead to increased antiviral efficacy. An exciting future prospect is to conjugate our lead compounds to lanthanide doped “upconversion” organic nanocrystals, which can absorb at deep tissue penetrating near infrared (NIR) wavelengths (>900 nm) and emit light at visible wavelengths [32], [33], [34]. The nitrogen on thiazolidine ring of LJ001 can tolerate many different substituents without loss of antiviral activity [4]; the nitrogen on the oxazolidine ring of JL118 and JL122 is likely suited for such conjugation purposes. Thus, an enhanced understanding of the precise molecular mechanism of action can guide the proper development of membrane-targeted photosensitizers as broad-spectrum antivirals.

Taken together, this study suggests that targeting the physiological differences between virus and cell membranes represents a novel therapeutic antiviral strategy worthy of further investigation. Another class of membrane targeted broad-spectrum antivirals (termed Rigid Amphipathic Fusion Inhibitors, RAFIs) was described shortly after our original publication of LJ001 by St Vincent *et al.* [5]. The authors reasonably contend that the “inverted-cone” shape of RAFIs (with respect to a larger hydrophilic headgroup) impairs the positive-to-negative curvature transition that is critical for productive membrane fusion, a well-known property of other inverted cone-shaped molecules such as lysophospholipids [35]. However, it is also hard to attribute the nanomolar antiviral activity of RAFIs entirely to their lipid binding properties and changes to their molecular geometry, given the molar excess of cellular membranes in any viral-cell infection assay [36], [37]. Although RAFIs are nucleoside derivatives with no chemical relation to LJ001 or the JL series of compounds, the hydrophobic group, perylene, present in *effective* RAFIs has a structure closely related to hypocrellin A, a well-known photosensitizer belonging to the family of quinones [36], [38]. It will be of interest to determine if the potential photosensitizing properties of active RAFIs could contribute to their antiviral activity.

In summary, thorough characterization of the mechanism of action and SAR optimization of LJ001 led to a new class of membrane-targeted photosensitizers (oxazolidine-2,4-dithiones) with increased potencies, ^1^O_2_ quantum yields, and red-shifted absorption spectra. Altogether, these improved properties resulted in membrane-targeted photosensitizers with encouraging *in vivo* antiviral efficacy against a lethal emerging pathogen. In light of our current study, the substantial literature on the *in vivo* use of photosensitizers [19] in the photodynamic therapy (PDT) of cancer should be re-examined for its applicability in the development of membrane-targeting broad-spectrum antivirals against lipid-enveloped viruses. Potentially, the most effective oxazolidine-2,4-dithiones could be evaluated as new candidate drugs in the photodynamic treatment of cancer.

## Materials and Methods

### Ethics statement - pharmacokinetics (PK) and animal challenge studies

All procedures and animal studies were in accordance with the National Research Council (NRC) *Guide for the Care and Use of Laboratory Animals* (1996) and/or approved by the Institutional Animal Care and Use Committee (IACUC) at the University of Texas Medical Branch (UTMB) and performed at the Robert E. Shope biosafety level 4 (BSL-4) laboratory. Methodological details and PK results are further provided in Text S1.

### Medicinal chemistry

The overall synthetic scheme for the JL series and structures of selected LJ and JL compounds are detailed in Text S1 and Figure S10, respectively. The absorbance spectra of compounds were determined on a monochromator-based Tecan Infinite M-1000 PRO by continuous scanning (λ = 250–800 nm) in absorbance mode using 100 µM of compound in 100 µl DMSO.

### Virological assays

Viral strains used, determination of IC_50_, and virus inhibition assays in the presence of red blood cells are indicated in Text S1. All assays were performed at or above biosafety levels corresponding to the risk group of the agents and NIH requirements.

### Reagents for membrane biophysical assays

Phospholipid species, liposome compositions, and fluorescent membrane probes are indicated in Text S1.

### Fluorescence spectroscopy measurements

Partition and acrylamide quenching studies were carried out using a Varian Cary Eclipse fluorescence spectrophotometer. Excitation and emission wavelength of LJ001 and LJ025 used were described in [4]. Excitation and emission spectra were corrected for wavelength-dependent instrumental factors [39], emission was also corrected for successive dilutions, light scattering [40] and simultaneous light absorption by quencher and fluorophore (inner filter effect).

### Partition coefficients determination

Membrane partition studies were performed with LUV by successive additions of small amounts of lipid systems, including pure POPC and HIV membrane-like mixture (POPC 5.3%, DPPC 3.5%, cholesterol 45.3%, SM 18.2%, POPE 19.3% and POPS 8.4%; mol % [15]), to 50 µM LJ001 or LJ025 solutions, with 10 min incubation between each addition. The partition coefficients (K_p_) were calculated from the fit of the experimental data with [41], [42]:
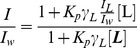
(1)where *I*_W_ and *I*_L_ are the fluorescence intensities in aqueous solution and in lipid, respectively, γ_L_ the molar volume of the lipid [43], and [L] the lipid concentration.

### Acrylamide quenching

Quenching of LJ001 or LJ025 by acrylamide [44] was studied in buffer and in the presence of POPC (LUV) as described elsewhere [44], [45] and in Figure S6.

### Changes on the surface pressure of lipid monolayers

The changes of the surface pressure of lipid monolayers induced by LJ001 or LJ025 were measured in a Langmuir-Blodgett trough ST900 at constant temperature (25.0±0.5°C). The surface of an HEPES buffer solution contained in the Teflon trough was exhaustively cleaned by aspiration. Then, a chloroform solution of lipids was spread on this surface to reach surface pressures between 22 and 29 mN/m. At each chosen surface pressure, molecules solutions were injected in the subphase and the changes on the surface pressure were followed during time to reach a constant value.

### Steady-state anisotropy measurement

3 mM LUV of POPC or HIV-like mixture prepared as described for partition assays were incubated with DPH or TMA-DPH to achieve a final probe concentration of 0.33 mol% (relative to the lipid). Steady-state anisotropy 〈r〉 was calculated using:
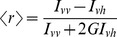
(4)where I_vv_ and I_vh_ represent the fluorescence intensities obtained with vertical excitation polarization and vertical or horizontal orientations of emission polarizers respectively. G = I_hv_/I_hh_ is a correction factor accounting for the polarization bias in the detection system. DPH probe: excitation 350 nm, emission 452 nm. TMA-DPH probe: excitation 355 nm, emission 430 nm.

Peripheral blood mononuclear cells (PBMC) obtained as described elsewhere [42] were incubated at 3×10^6^ cells/ml in buffer with 2.5 µM of DPH or TMA-DPH, during 30 min, with gentle stirring. The <*r*> values obtained for control PBMC using DPH and TMA-DPH (0.302±0.016 and 0.317±0.055, respectively) are in a good agreement with reference values obtained in a previous works [46]. Fluorescently labeled PBMC were then incubated with LJ001 or LJ025 during 1 h, with gentle agitation, before the fluorescence anisotropy measurements, conducted as indicated above.

### Singlet oxygen (^1^O_2_) production and quenching by the JL series

^1^O_2_ quantum yields (QY) and quenching rate constants were determined using a time-resolved set-up (Nd∶YAG Minilase II, New Wave Research Inc.), excitation pulse duration 4–6 ns at 355 nm and 5–7 ns at 532 nm, and a liquid N_2_ cooled Ge photodetector (Applied Detector Corporation Model 403 S). Details of the filters used have been described elsewhere [47]. Signals were digitized on a LeCroy 9350 CM 500 MHz oscilloscope and analyzed using Origin software. All experiments were carried out at ambient temperature and in air-saturated solutions. UV-visible spectra were recorded on a Cary 300 Bio Spectrophotometer (Varian).

### Singlet oxygen quantum yield measurements

Samples were prepared in deuterated methylene chloride (CD_2_Cl_2_) with absorbances between 0.04–0.3 at 355 nm or 532 nm. The laser pulse energy was 1–2.5 mJ at 355 nm and 3–4 mJ at 532 nm. The absorbance of the reference sensitizer (Rose Bengal, TPP and C_60_) and the series compounds were matched within 80%. The initial ^1^O_2_ intensity was extrapolated to t = 0. Data points of the initial 0–5 µs were not used due to electronic interference signals from the detector.

### Singlet oxygen quenching measurements

The quenching rates (*k*_T_) of ^1^O_2_ were measured by Stern–Volmer analysis using C_60_ as sensitizer at 355 nm in methylene chloride. Concentration of the samples used in the measurements ranged between 0.01–1 mM.

### Lipid oxidation and viral lipodomics

Briefly, lipid oxidation in recombinant unilamellar liposomes (7∶3 molar ratio of phosphatidylcholine∶cholesterol, >60% linoleic acid) untreated or treated with 10 µM compounds and light was determined on extracted lipids by LC-MS/MS analysis, as previously described [48]. The transitions monitored were mass-to-charge ratio (m/z): m/z 295→194.8 for 13-HODE; 295→171 for 9-HODE; and 299→197.9 for 13-HODE-d_4_. Methodological details are further provided in Text S1. Viral lipidome analysis was performed on lipids extracted from Influenza A virus (A/PR/8/34 H1N1) treated with 5 µM of LJ001 or the negative control LJ025, exposed to light for 1 h as described [49], [50].

## Supporting Information

Figure S1 **Schematic for the HIV time-of-addition experiment and fusion cascade (Class I).** The inhibition half-lives (*t*_1/2_) for anti-CD4 (leu3A), T-20, and LJ001 are taken from the data presented in Figure 1A. Inset shows how the T-20 peptide is thought to inhibit the transition from the prehairpin intermediate (PHI) to the 6-helix bundle (6-HB).
(PDF)

Figure S2 **Antiviral activity of LJ001 against Semliki Forest virus (SFV).** SFV was treated with increasing concentrations of LJ001, under identical light exposure conditions as described in Materials and Methods, and used to infect target BHK cells. Following infection for 1.5 h, cells were incubated at 28°C overnight in media containing 20 mM NH_4_Cl to prevent secondary infection. Infected cells were quantified by immunofluorescence [52], and results are presented as % of infection (mean ± SD, n = 3) relative to that obtained in the absence of LJ001 treatment.
(PDF)

Figure S3 **Lipidome analysis of LJ001-treated purified influenza A virus (A/PR/8/34 H1N1).** Influenza virus was treated with 5 µM of LJ001 or the negative control LJ025, exposed to light for 1 h, and subsequently subjected to lipid extraction. Analyses of lipids, including oxidized species, were carried out using a high-resolution Thermo LTQ-Orbitap mass spectrometer and an ABI 3200 QTRAP mass spectrometer after liquid chromatography separation [49], [50]. Similar results were obtained in two independent experiments and data is represented as a single stage positive ion mass spectrum (over a m/z range of 1 Da). The hydroperoxide (OO)PC 36∶2 is shown as an example of the prominent changes in Figure 2C. The precision of our measurements (Δ<1 ppm) allow us to distinguish the spectrum of oxidized (OO)PC 36∶2 (m/z = 818.5910) from (unoxidized) ePC 40∶6 (m/z = 818.6063). The former is present in the LJ001 treated sample, but almost completely absent in the LJ025 sample.
(PDF)

Figure S4 **LJ001-mediated oxidation of DMA.** LJ001, the inactive control LJ025 or the positive control methylene blue (MB) were added to a solution of DMA and exposed to light. At 0.1, 1, 3 or 6 h, DMA conversion was detected by ^1^H-NMR (DMA∶oxiDMA = 3.1 ppm:2.1 ppm (methyl peak)). Reactions were performed in CDCl_3_ using 1 equivalent of each reagent (DMA, sensitizer and α-tocopherol, where applicable). CDCl_3_ was saturated with oxygen (O_2_) by bubbling O_2_ through the solvent for 30 min and the reaction was kept under O_2_ gas atmosphere, except for “Ar” where oxygen was exchanged with argon by the freeze/thaw method. Data represents the mean ± SD of duplicate experiments.
(PDF)

Figure S5 **Time-resolved singlet-oxygen phosphorescence trace.** The singlet-oxygen phosphorescence trace was recorded at 1270 nm from a solution of LJ001 in air-saturated deuterated methylene chloride (CD_2_Cl_2_) pulsed with a Nd∶YAG laser at 355 nm.
(PDF)

Figure S6
**Stern-Volmer plots for the quenching of LJ001 and LJ025 fluorescence in 3 mM POPC vesicles by acrylamide (water-soluble, and excluded from the interior of the membrane).** Each point is the average of three independent measures. Error bars indicate standard deviations. Quenching of 50 µM LJ001 or LJ025 by acrylamide (0–60 mM) was studied in buffer and in the presence of POPC 3 mM (LUV), by successive additions of small volumes of the quencher stock solution [44]. For every addition, a minimal 10 min incubation time was allowed before measurement. Quenching data were analyzed by using the Stern–Volmer equation [41];
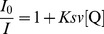
(2)or the Lehrer equation [53], [54], [55], when a negative deviation to the Stern–Volmer relationship was observed:
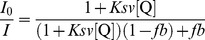
(3)where *I* and *I*_0_ are the fluorescence intensities of the sample in the presence and absence of quencher, respectively, *K*_SV_ is the Stern–Volmer constant, [*Q*] is the concentration of quencher, and *f_b_* the fraction of light emitted by the molecules accessible to the quencher.(PDF)

Figure S7 **Schematic representation of the effect of singlet oxygen (^1^O_2_) generated by LJ001 on the phospholipids composing a viral membrane.** From top to bottom row: (***Fatty acid***) *Trans*-isomerization of linoleic acid after ^1^O_2_ attack on C13 following the “ene” reaction. The oxidation results in a hydroperoxide (HpODE) intermediate ultimately reduced into a hydroxyl octadecadienoic (HODE) acid. (***Phospholipid***) The *trans*-isomerization of a linoleic acid chain of a 36∶2 phospholipid results in a decreased overall diameter of the phospholipid species and insertion into the highly hydrophobic chain of a polar (less hydrophobic) group. Both the HpODE intermediate and final HODE are represented underneath their corresponding formula drawing. (***Membrane***) the reduction of the diameter of the 36∶2 phospholipid results in a tighter packing of the phospholipids composing the membrane. Repulsion of the more polar lateral chains also results in a clustering of the oxidized lipids (in microdomains). (***Virus***) At the scale of the virus, the shrinkage of the particle diameter due to tighter packing of the *trans*-isomerized unsaturated phospholipids may result in increased positive curvature, while the clustering of the oxidized lipids will result in decreased membrane fluidity. Thus, ^1^O_2_-mediated lipid oxidation results in changes in the biophysical properties of the viral membrane that negatively impacts on its ability to undergo virus-cell membrane fusion (see [36], [56]).
(PDF)

Figure S8 **Comparative antiviral activity of LJ001 and JL103.** The antiviral activity of LJ001 and JL103 were determined for the indicated viruses representing all three classes of viral fusion proteins (Figure 5B). Full dose response experiments were carried at multiplicities of infection (MOIs) within the linear range or at dilutions compatible with plaque assay studies. All viruses were incubated with serial dilutions of LJ001 or JL103 in clear eppendorf tubes, which were exposed for 10 min to the white fluorescent light of the biosafety cabinet (BSC) at room temperature, before infecting the target cells. To maximize light exposure, eppendorf tubes were laid flat on the BSC working surface during the 10 min light exposure. At the appropriate time post-infection, the percent of infection was evaluated according to the assay corresponding to the virus under study (see Materials and Methods). The maximum relative infection, 100%, was set for the untreated control. Data shown here are the average (± SD) or representative graphs of 2–6 independent repeats. Data were plotted and analyzed using GraphPad PRISM software and the IC_50_ were calculated by non-linear regression analysis with variable slopes with constraints set for the maximum and minimum at respectively 100 and 0%. Viruses with Class I fusion proteins: **HIV**: human immunodeficiency virus-1 JRCSF (R5-tropic); **NDV**: Newcastle disease virus; **HeV**: Hendra virus; **NiV**: Nipah virus Malaysia; **H1N1**: Influenza A A/PR/8/34 (H1N1); **EBOV**: Ebola Zaire. Viruses with Class II fusion proteins: **RVFV**: Rift Valley fever MP-12 (vaccine strain); **SFV**: Semliki forest virus. Viruses with Class III fusion proteins: **VSV**: Vesicular stomatitis virus; **CMV**: Cytomegalovirus (strain T3259); **HSV**: Herpes simplex virus-1; **RABV**: Rabies virus. Non-enveloped virus: **Ad5**: Adenovirus serotype 5.
(PDF)

Figure S9 **The antiviral activity of JL103 is dependent on light.** (**A**) HIV, HSV or NDV were treated in the dark with 1 µM of JL103 and subsequently either exposed to the white light source of the BSC or kept in the dark for 10 min before infection of cells in the dark (see Materials and Methods). Infection as determined by luciferase activity (HIV) or GFP expression by flow cytometry (HSV and NDV) is reported relative to untreated virus (100%). Note that the bars representing LJ001-treated viruses exposed to light cannot be seen in the figure and represent at least 99% reduction in infectivity. Data represents the mean ± SD of duplicate experiments. (**B**) HIV-1_IIIB_ was incubated with 6.25 nM of JL103 in the presence of α-tocopherol or DMA (serial 2-fold dilutions from 100 to 3.125 µM). Infection of TZM-bl cells was determined by luciferase activity in cell lysates 48 h post-infection and is reported relative to untreated virus (100%). Data represents the mean ± SD of duplicate experiments. (**C**) HIV-1_JR-CSF_ infection was synchronized by spinoculation of the virus for 2 h at 4°C on reporter TZM-BL cells. The plates were subsequently shifted to room temperature (t = 0) for 1 h before incubating at 37°C. LJ001 (20 µM), JL103 (20 µM), HIV entry inhibitors specifically blocking CD4-attachment (Leu-3A, 10 µg/ml) or 6-HB formation (T-20, 5 µM)), or the reverse transcriptase inhibitor AZT (20 µM) were added at 0, 15, 30, 60, 75, 90 and 120 min post-spinoculation. Luciferase expression in cell lysates was analyzed 48 h post-infection and expressed relative to untreated control (100%). Data representing the mean ± SD of duplicate experiments were graphed, and *t*_1/2_ values calculated using GraphPad PRISM. Due to the higher efficiency of JL103 to inhibit viral entry and the conditions of our assay (see Figure S1), where the fusion permissive conditions were extended at suboptimal temperatures, we cannot be sure that that all viruses have fused by the 2-hour time point, hence the partial inhibition still observed at 2 h for JL103. (**D**) LJ001, JL102 or JL103 were added to a solution of DMA and exposed to light. At 0.1, 1, 3 or 6 h, DMA conversion was detected by ^1^H-NMR (DMA∶oxiDMA = 3.1 ppm:2.1 ppm (methyl peak)). Reactions were performed in CDCl_3_ using 1 equivalent of each reagent (DMA, sensitizer and α-tocopherol, where applicable). CDCl_3_ was saturated with oxygen (O_2_) by bubbling O_2_ through the solvent for 30 min and the reaction was kept under O_2_ gas atmosphere, except for Ar where oxygen was exchanged with argon by freeze/thaw method. Data represents the mean ± SD of duplicate experiments.
(PDF)

Figure S10 **Structures of selected LJ and JL-series compounds.** All stock solutions of compounds were in DMSO at a final concentration of 10 mM.
(PDF)

Figure S11 **Absorbance spectra of selected oxazolidine dithiones.** The indicated compounds were dissolved in 100 µl DMSO to a final concentration of 100 µM, and the absorbance scan done using Tecan Infinite M-1000 PRO plate reader.
(PDF)

Figure S12 **Post-exposure** ***in vivo*** **efficacy of JL103 in a lethal challenge model of Rift valley fever virus (RVFV).** (**A**) Balb/c mice were challenged intraperitoneally (IP) with 1 or 20 pfu (plaque forming units) of RVFV ZH501. Mice were monitored daily and survival as a Kaplan-Meier plot was compared with the Log-rank (Mantel-Cox) test using GraphPad PRISM to obtain the LD_50_. (**B**) Balb/c mice, lethally challenged IP with 20 pfu of RVFV, were left untreated or treated IP once a day for 7 days, starting 1 h post-challenge, with JL103 (10 mg/kg). Mice were monitored daily and survival as a Kaplan-Meier plot was compared with the Log-rank (Mantel-Cox) test using GraphPad PRISM to determine the median time-to-death.
(PDF)

Table S1 **Parameters obtained from the fitting of the fluorescence data of partition assays of selected 2-(thio)oxothiazolidin-4-ones (LJ001 and LJ025) and oxazolidine dithiones (JL103, JL118 and JL122).**
(PDF)

Table S2 **Photophysical properties of selected 2-(thio)oxothiazolidin-4-ones (LJ001 and LJ025) and oxazolidine dithiones (JL102-122).**
(PDF)

Table S3 **Antiviral activity, cytotoxicity, and therapeutic indexes of selected 2-(thio)oxothiazolidin-4-ones (LJ001 and LJ025) and oxazolidine dithiones (JL101-JL122).**
(PDF)

Table S4 **Pharmacokinetics of selected 2-(thio)oxothiazolidin-4-one (LJ001) and oxazolidine dithiones (JL103, JL118, JL122).**
(PDF)

Text S1 **Supporting information.** Supporting Materials and Methods.
(DOC)
