# Supplementary material for: A Mechanistic Paradigm for Broad-Spectrum Antivirals that Target Virus-Cell Fusion
Source: PLoS Pathog. 2013 Apr 18;9(4):e1003297. doi: 10.1371/journal.ppat.1003297 (PMC3630091; doi:10.1371/journal.ppat.1003297)
Supplement: Figure S1 — Schematic for the HIV time-of-addition experiment and fusion cascade (Class I). The inhibition half-lives (t 1/2) for anti-CD4 (leu3A), T-20, and LJ001 are taken from the data presented in Figure 1A. Inset shows how the T-20 peptide is thought to inhibit the transition from the prehairpin intermediate (PHI) to the 6-helix bundle (6-HB). (PDF) [file ppat.1003297.s001.pdf]

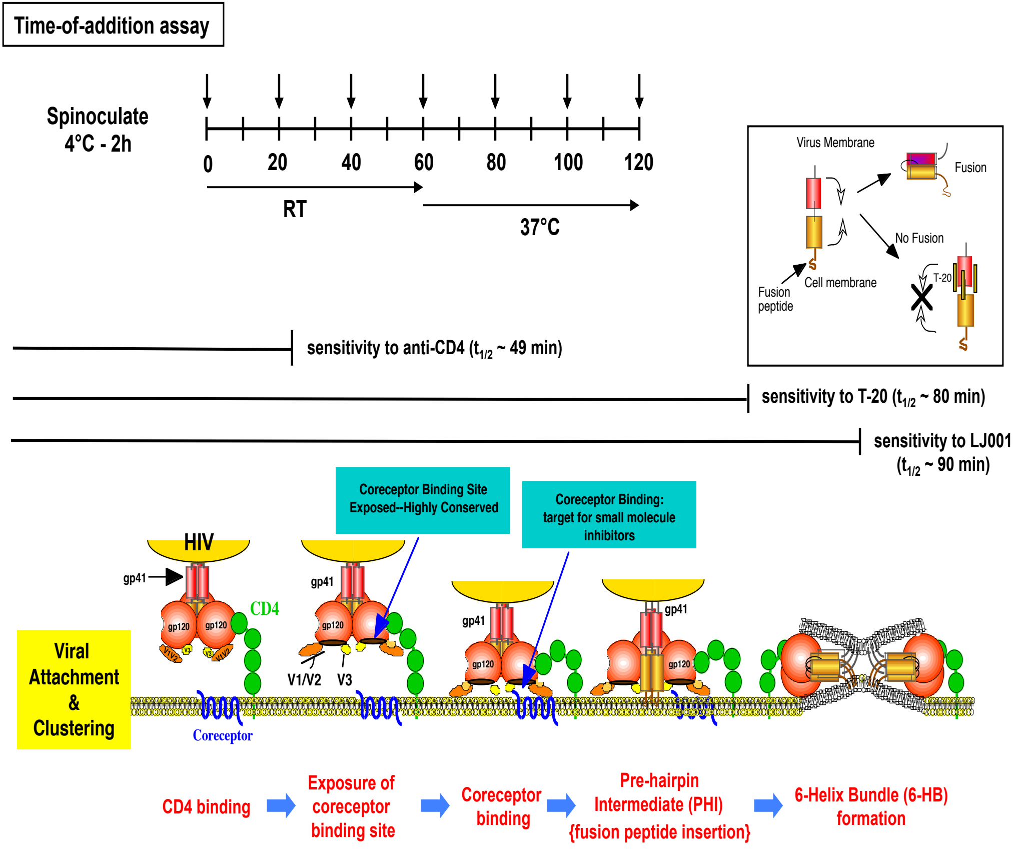
Time-of-addition assay
Spinoculate
4°C - 2h
0
20
40
60
80
100
120
RT
37°C
Virus Membrane
Fusion
No Fusion
T-20
Cell membrane
Fusion
peptide
sensitivity to anti-CD4 ($t_{1/2}$ ~ 49 min)
sensitivity to T-20 ($t_{1/2}$ ~ 80 min)
sensitivity to LJ001
($t_{1/2}$ ~ 90 min)
Coreceptor Binding Site
Exposed--Highly Conserved
Coreceptor Binding:
target for small molecule
inhibitors
HIV
gp41
gp120
CD4
V1/V2
V3
Coreceptor
Viral
Attachment
&
Clustering
CD4 binding
Exposure of
coreceptor
binding site
Coreceptor
binding
Pre-hairpin
Intermediate (PHI)
{fusion peptide insertion}
6-Helix Bundle (6-HB)
formation
