# Supplementary figures and images for: A Mechanistic Paradigm for Broad-Spectrum Antivirals that Target Virus-Cell Fusion
Source: PLoS Pathog. 2013 Apr 18;9(4):e1003297. doi: 10.1371/journal.ppat.1003297 (PMC3630091; doi:10.1371/journal.ppat.1003297)

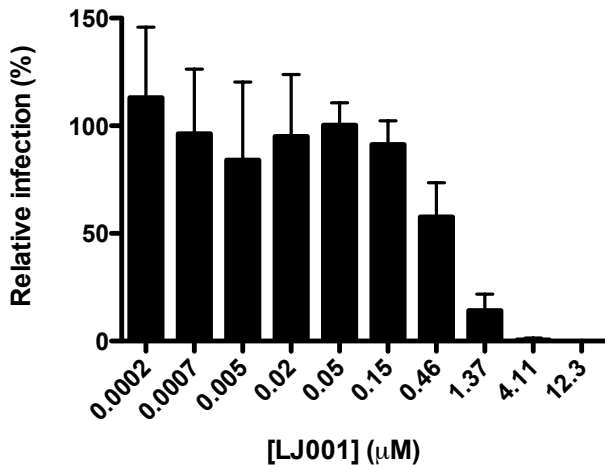

Supplement: Figure S2 — Antiviral activity of LJ001 against Semliki Forest virus (SFV). SFV was treated with increasing concentrations of LJ001, under identical light exposure conditions as described in Materials and Methods, and used to infect target BHK cells. Following infection for 1.5 h, cells were incubated at 28°C overnight in media containing 20 mM NH4Cl to prevent secondary infection. Infected cells were quantified by immunofluorescence [52], and results are presented as % of infection (mean ± SD, n = 3) relative to that obtained in the absence of LJ001 treatment. (PDF) [file ppat.1003297.s002.pdf]

**LJ025**

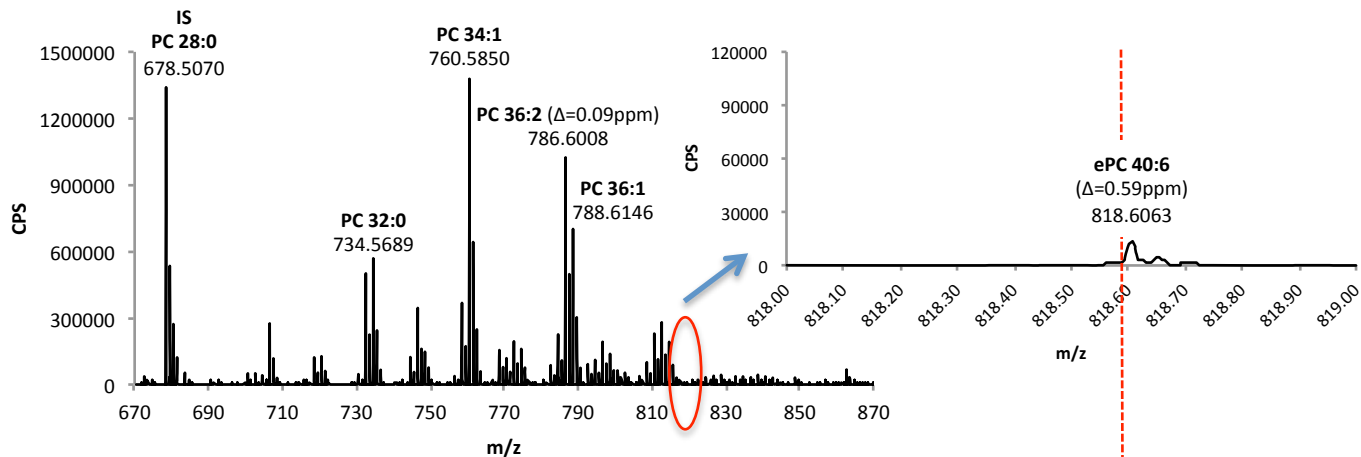

**LJ001**

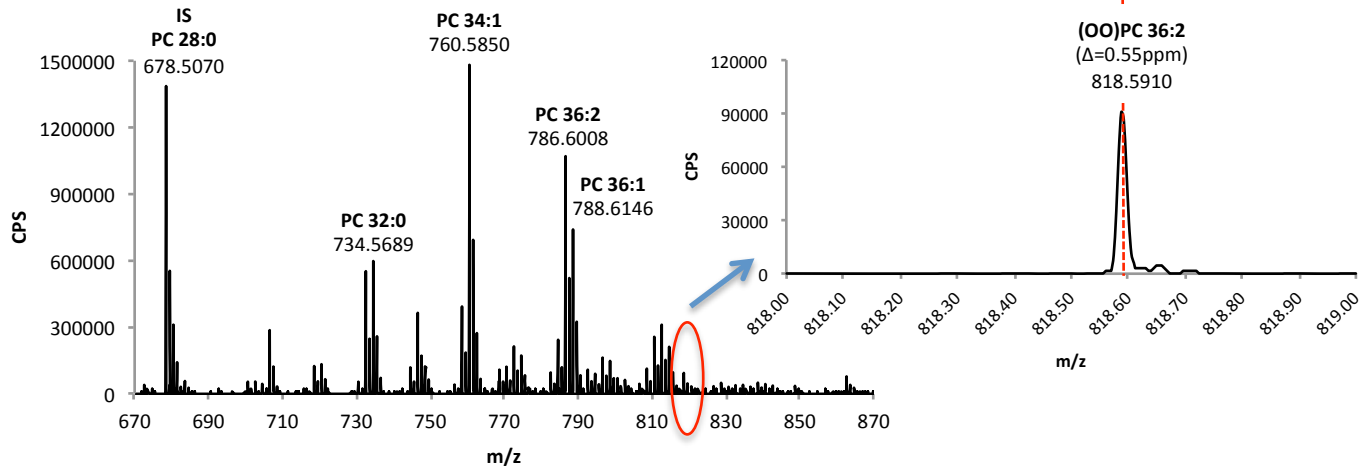

Supplement: Figure S3 — Lipidome analysis of LJ001-treated purified influenza A virus (A/PR/8/34 H1N1). Influenza virus was treated with 5 µM of LJ001 or the negative control LJ025, exposed to light for 1 h, and subsequently subjected to lipid extraction. Analyses of lipids, including oxidized species, were carried out using a high-resolution Thermo LTQ-Orbitap mass spectrometer and an ABI 3200 QTRAP mass spectrometer after liquid chromatography separation [49], [50]. Similar results were obtained in two independent experiments and data is represented as a single stage positive ion mass spectrum (over a m/z range of 1 Da). The hydroperoxide (OO)PC 36∶2 is shown as an example of the prominent changes in Figure 2C. The precision of our measurements (Δ<1 ppm) allow us to distinguish the spectrum of oxidized (OO)PC 36∶2 (m/z = 818.5910) from (unoxidized) ePC 40∶6 (m/z = 818.6063). The former is present in the LJ001 treated sample, but almost completely absent in the LJ025 sample. (PDF) [file ppat.1003297.s003.pdf]

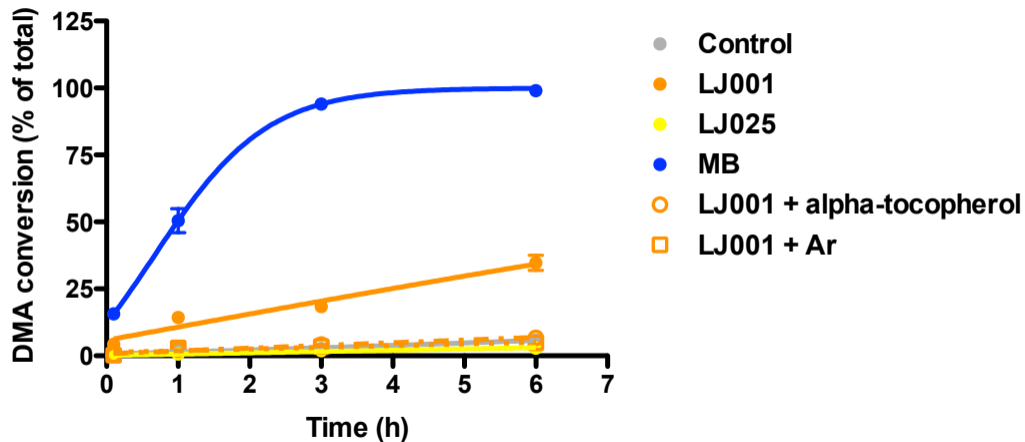

Supplement: Figure S4 — LJ001-mediated oxidation of DMA. LJ001, the inactive control LJ025 or the positive control methylene blue (MB) were added to a solution of DMA and exposed to light. At 0.1, 1, 3 or 6 h, DMA conversion was detected by 1H-NMR (DMA∶oxiDMA = 3.1 ppm:2.1 ppm (methyl peak)). Reactions were performed in CDCl3 using 1 equivalent of each reagent (DMA, sensitizer and α-tocopherol, where applicable). CDCl3 was saturated with oxygen (O2) by bubbling O2 through the solvent for 30 min and the reaction was kept under O2 gas atmosphere, except for “Ar” where oxygen was exchanged with argon by the freeze/thaw method. Data represents the mean ± SD of duplicate experiments. (PDF) [file ppat.1003297.s004.pdf]

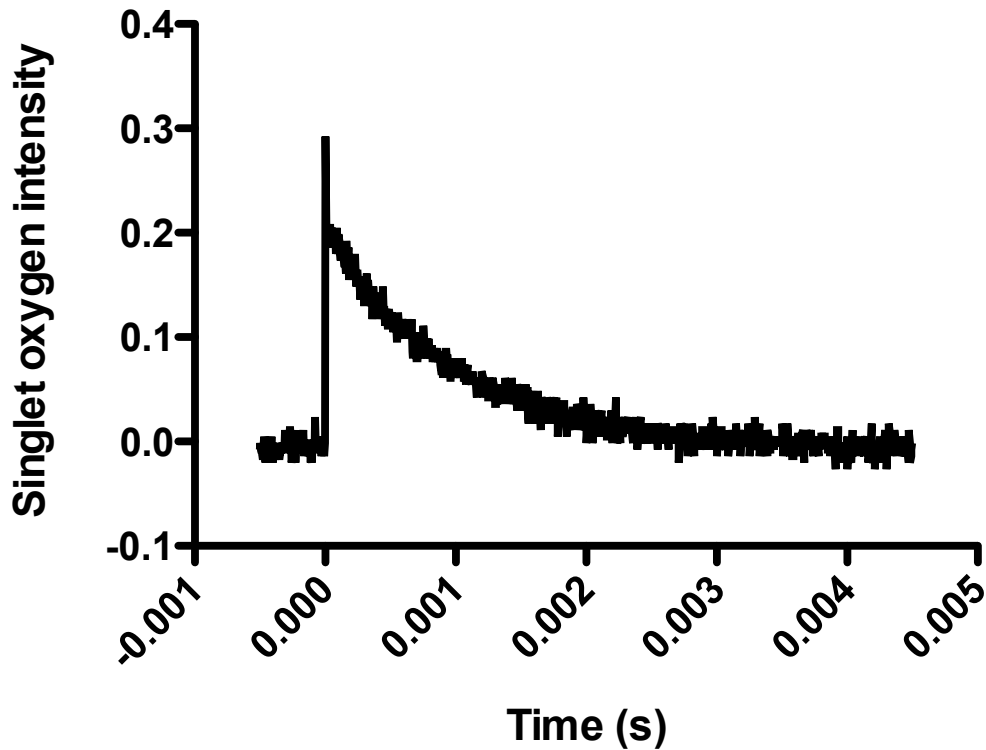

Supplement: Figure S5 — Time-resolved singlet-oxygen phosphorescence trace. The singlet-oxygen phosphorescence trace was recorded at 1270 nm from a solution of LJ001 in air-saturated deuterated methylene chloride (CD2Cl2) pulsed with a Nd∶YAG laser at 355 nm. (PDF) [file ppat.1003297.s005.pdf]

LJ001

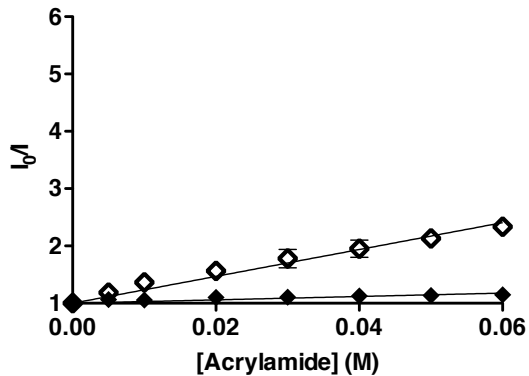

LJ025

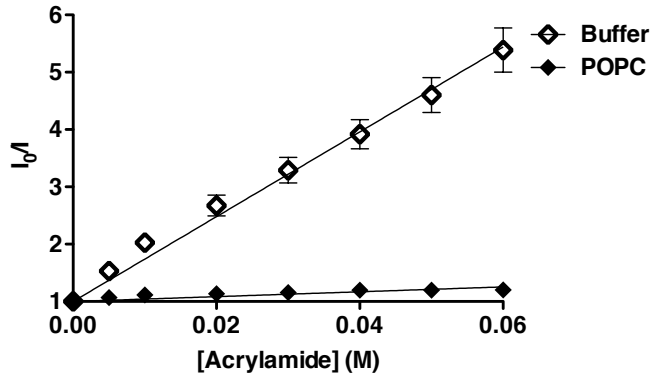

Supplement: Figure S6 — Stern-Volmer plots for the quenching of LJ001 and LJ025 fluorescence in 3 mM POPC vesicles by acrylamide (water-soluble, and excluded from the interior of the membrane). Each point is the average of three independent measures. Error bars indicate standard deviations. Quenching of 50 µM LJ001 or LJ025 by acrylamide (0–60 mM) was studied in buffer and in the presence of POPC 3 mM (LUV), by successive additions of small volumes of the quencher stock solution [44]. For every addition, a minimal 10 min incubation time was allowed before measurement. Quenching data were analyzed by using the Stern–Volmer equation [41];(2)or the Lehrer equation [53], [54], [55], when a negative deviation to the Stern–Volmer relationship was observed:(3)where I and I 0 are the fluorescence intensities of the sample in the presence and absence of quencher, respectively, K SV is the Stern–Volmer constant, [Q] is the concentration of quencher, and fb the fraction of light emitted by the molecules accessible to the quencher. (PDF) [file ppat.1003297.s006.pdf]

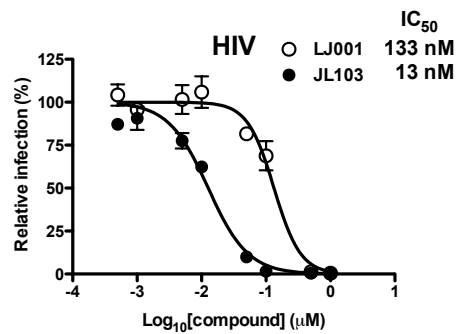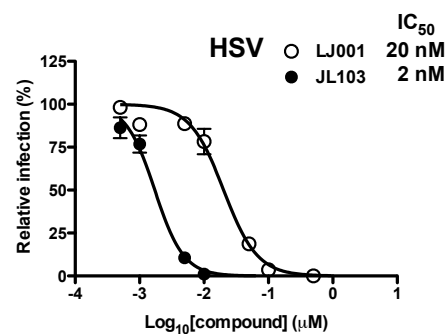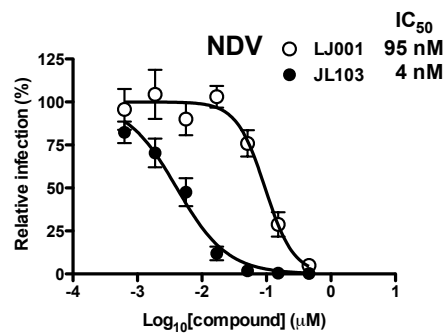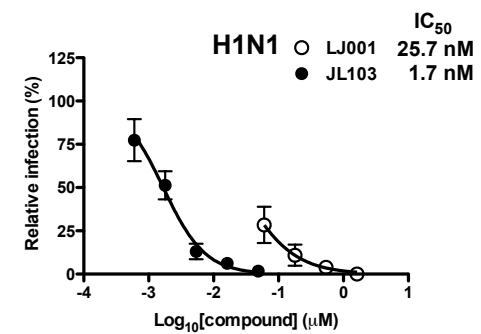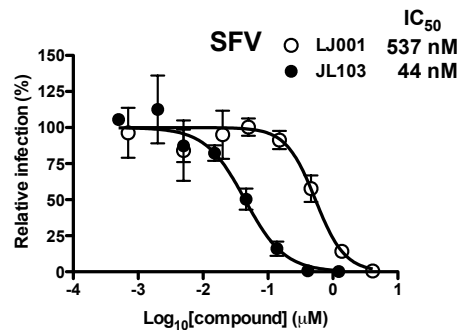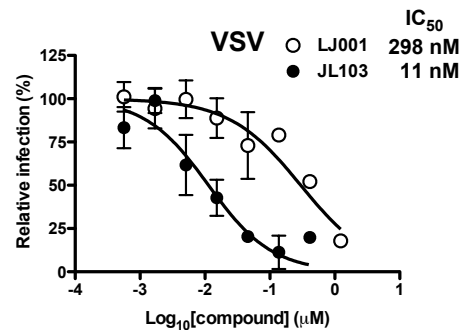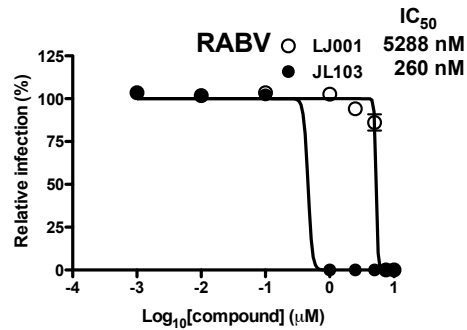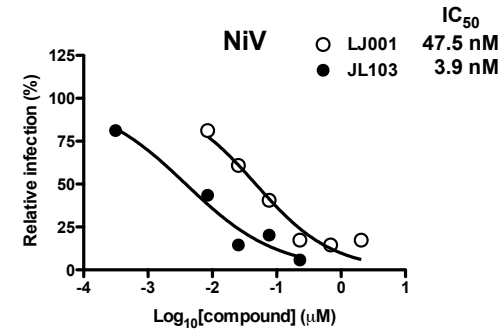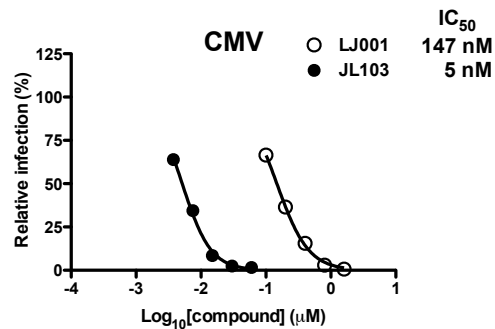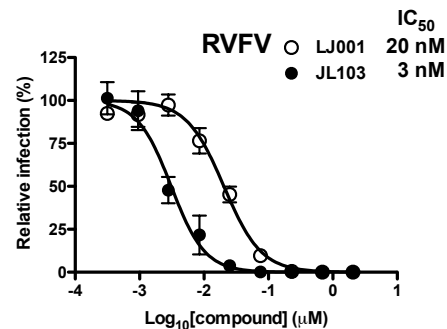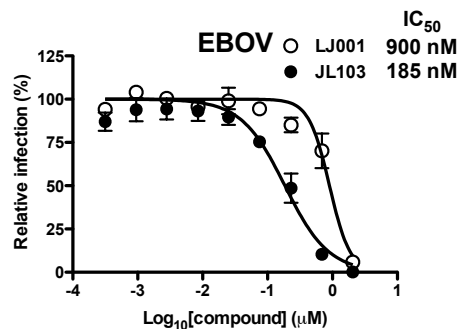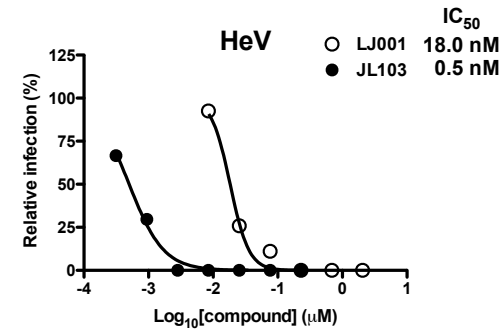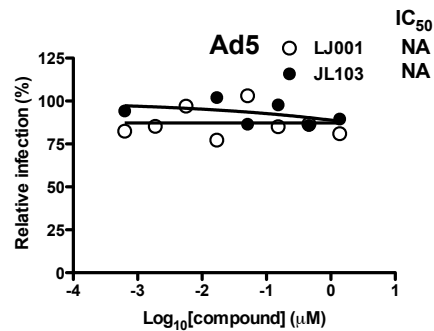

Supplement: Figure S8 — Comparative antiviral activity of LJ001 and JL103. The antiviral activity of LJ001 and JL103 were determined for the indicated viruses representing all three classes of viral fusion proteins (Figure 5B). Full dose response experiments were carried at multiplicities of infection (MOIs) within the linear range or at dilutions compatible with plaque assay studies. All viruses were incubated with serial dilutions of LJ001 or JL103 in clear eppendorf tubes, which were exposed for 10 min to the white fluorescent light of the biosafety cabinet (BSC) at room temperature, before infecting the target cells. To maximize light exposure, eppendorf tubes were laid flat on the BSC working surface during the 10 min light exposure. At the appropriate time post-infection, the percent of infection was evaluated according to the assay corresponding to the virus under study (see Materials and Methods). The maximum relative infection, 100%, was set for the untreated control. Data shown here are the average (± SD) or representative graphs of 2–6 independent repeats. Data were plotted and analyzed using GraphPad PRISM software and the IC50 were calculated by non-linear regression analysis with variable slopes with constraints set for the maximum and minimum at respectively 100 and 0%. Viruses with Class I fusion proteins: HIV: human immunodeficiency virus-1 JRCSF (R5-tropic); NDV: Newcastle disease virus; HeV: Hendra virus; NiV: Nipah virus Malaysia; H1N1: Influenza A A/PR/8/34 (H1N1); EBOV: Ebola Zaire. Viruses with Class II fusion proteins: RVFV: Rift Valley fever MP-12 (vaccine strain); SFV: Semliki forest virus. Viruses with Class III fusion proteins: VSV: Vesicular stomatitis virus; CMV: Cytomegalovirus (strain T3259); HSV: Herpes simplex virus-1; RABV: Rabies virus. Non-enveloped virus: Ad5: Adenovirus serotype 5. (PDF) [file ppat.1003297.s008.pdf]

**A**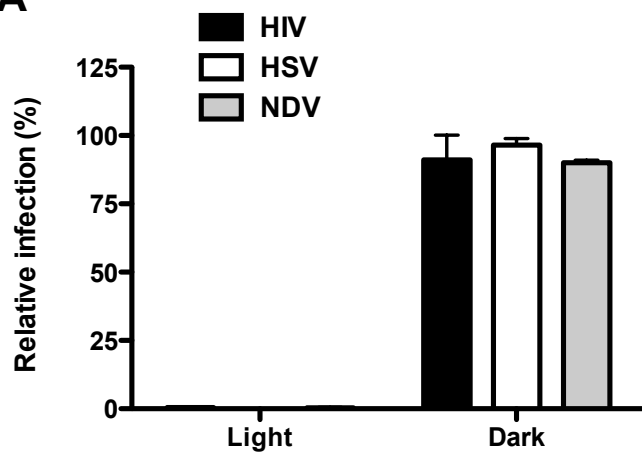**B**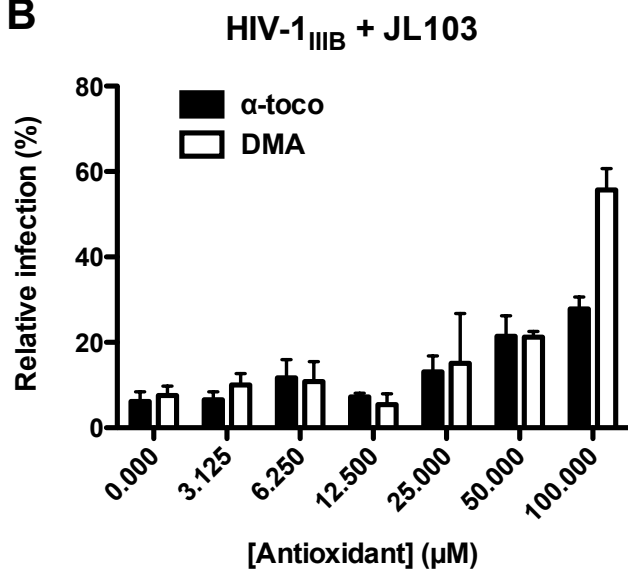**C**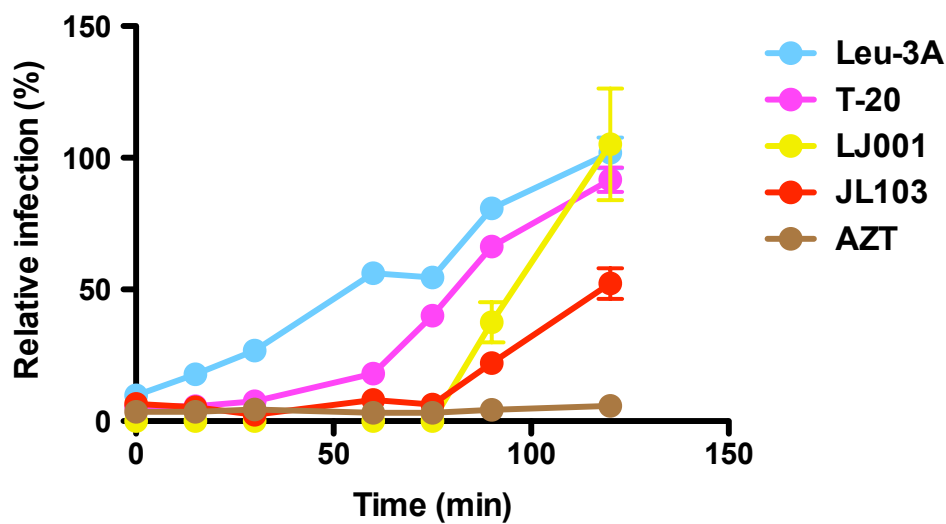**D**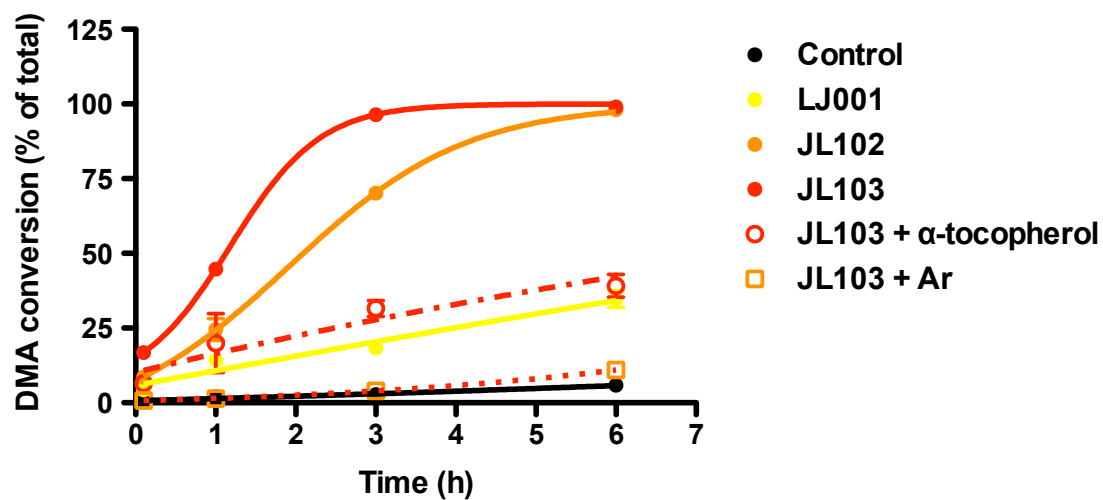

Supplement: Figure S9 — The antiviral activity of JL103 is dependent on light. (A) HIV, HSV or NDV were treated in the dark with 1 µM of JL103 and subsequently either exposed to the white light source of the BSC or kept in the dark for 10 min before infection of cells in the dark (see Materials and Methods). Infection as determined by luciferase activity (HIV) or GFP expression by flow cytometry (HSV and NDV) is reported relative to untreated virus (100%). Note that the bars representing LJ001-treated viruses exposed to light cannot be seen in the figure and represent at least 99% reduction in infectivity. Data represents the mean ± SD of duplicate experiments. (B) HIV-1IIIB was incubated with 6.25 nM of JL103 in the presence of α-tocopherol or DMA (serial 2-fold dilutions from 100 to 3.125 µM). Infection of TZM-bl cells was determined by luciferase activity in cell lysates 48 h post-infection and is reported relative to untreated virus (100%). Data represents the mean ± SD of duplicate experiments. (C) HIV-1JR-CSF infection was synchronized by spinoculation of the virus for 2 h at 4°C on reporter TZM-BL cells. The plates were subsequently shifted to room temperature (t = 0) for 1 h before incubating at 37°C. LJ001 (20 µM), JL103 (20 µM), HIV entry inhibitors specifically blocking CD4-attachment (Leu-3A, 10 µg/ml) or 6-HB formation (T-20, 5 µM)), or the reverse transcriptase inhibitor AZT (20 µM) were added at 0, 15, 30, 60, 75, 90 and 120 min post-spinoculation. Luciferase expression in cell lysates was analyzed 48 h post-infection and expressed relative to untreated control (100%). Data representing the mean ± SD of duplicate experiments were graphed, and t 1/2 values calculated using GraphPad PRISM. Due to the higher efficiency of JL103 to inhibit viral entry and the conditions of our assay (see Figure S1), where the fusion permissive conditions were extended at suboptimal temperatures, we cannot be sure that that all viruses have fused by the 2-hour time point, hence the partial inhibi [file ppat.1003297.s009.pdf]

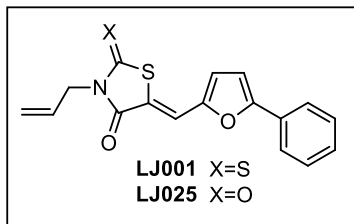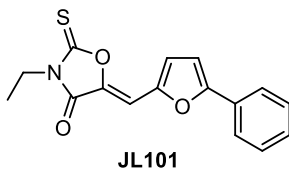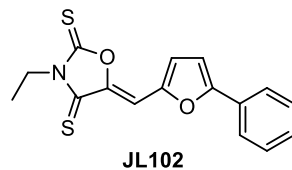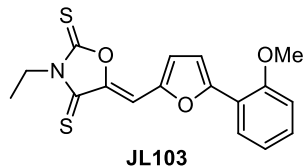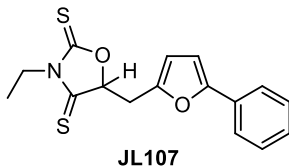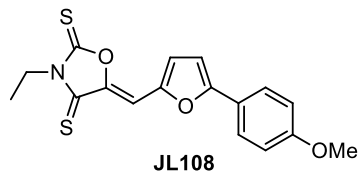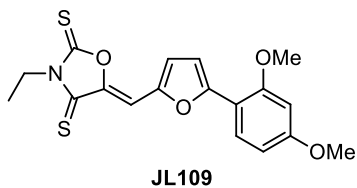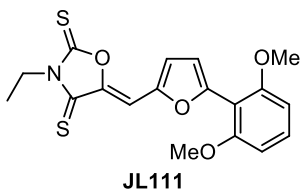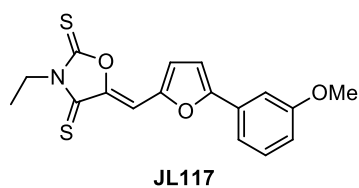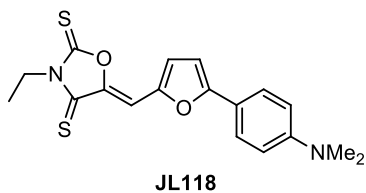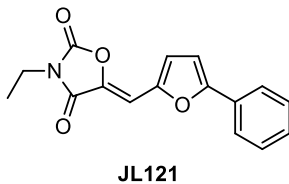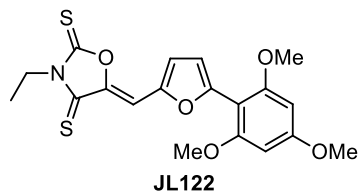

Supplement: Figure S10 — Structures of selected LJ and JL-series compounds. All stock solutions of compounds were in DMSO at a final concentration of 10 mM. (PDF) [file ppat.1003297.s010.pdf]

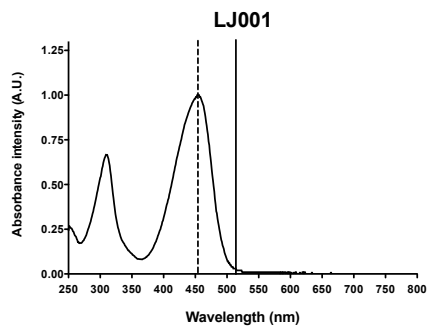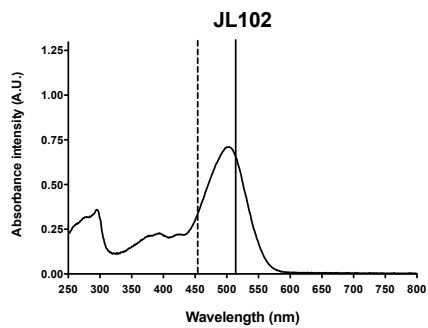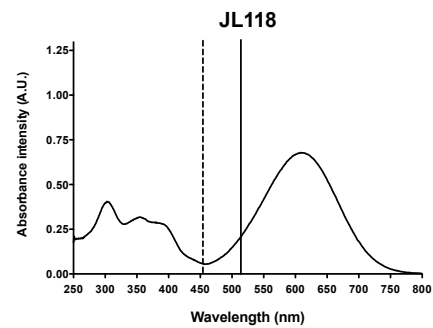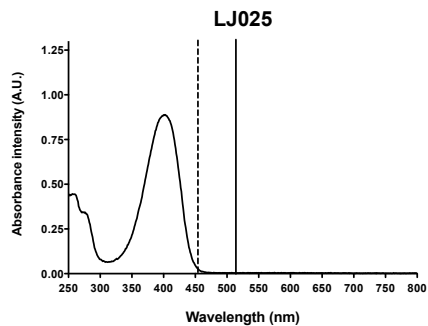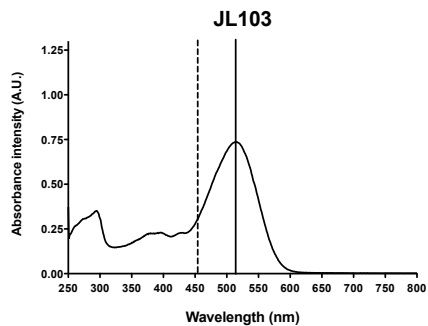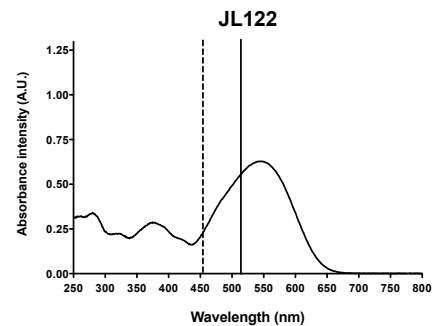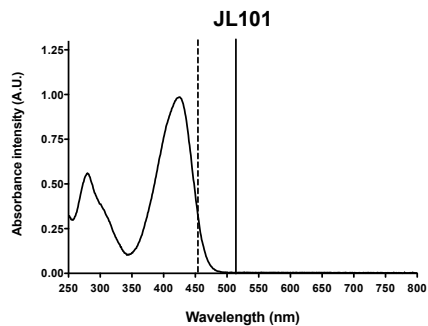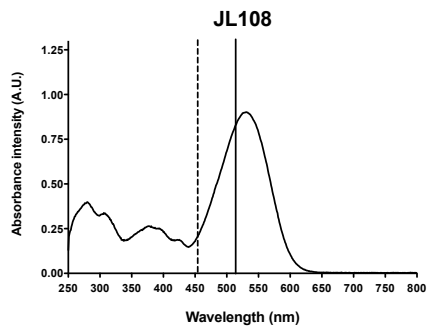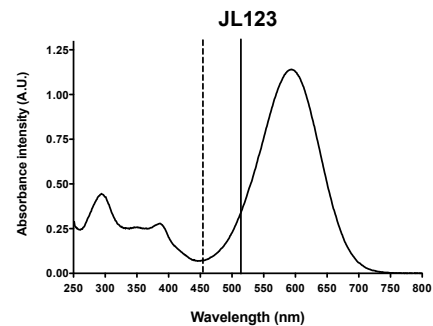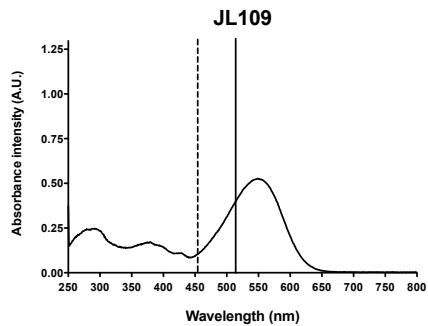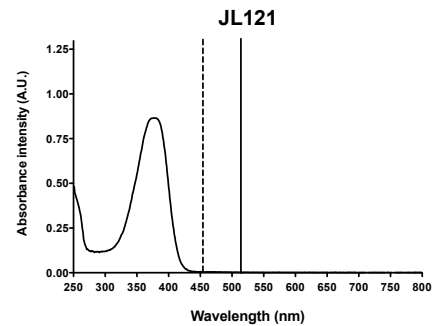

Supplement: Figure S11 — Absorbance spectra of selected oxazolidine dithiones. The indicated compounds were dissolved in 100 µl DMSO to a final concentration of 100 µM, and the absorbance scan done using Tecan Infinite M-1000 PRO plate reader. (PDF) [file ppat.1003297.s011.pdf]

**A****RVFV challenge**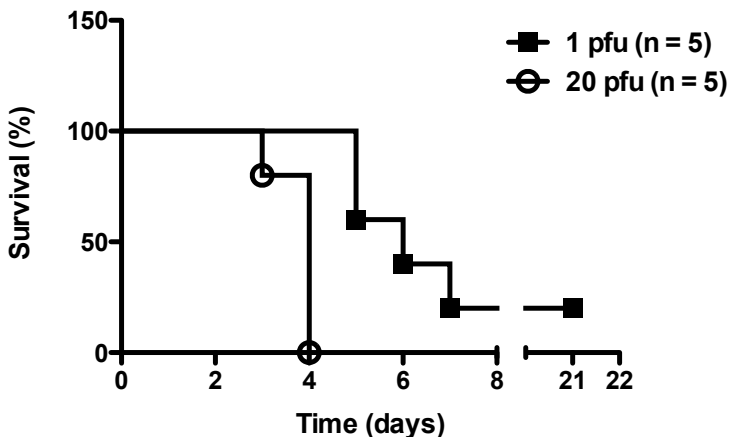**B****JL103 vs VIRUS - 20 pfu**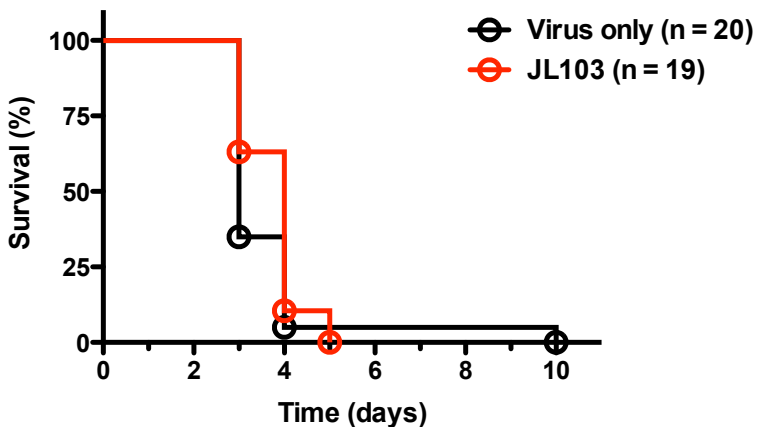

Supplement: Figure S12 — Post-exposure in vivo efficacy of JL103 in a lethal challenge model of Rift valley fever virus (RVFV). (A) Balb/c mice were challenged intraperitoneally (IP) with 1 or 20 pfu (plaque forming units) of RVFV ZH501. Mice were monitored daily and survival as a Kaplan-Meier plot was compared with the Log-rank (Mantel-Cox) test using GraphPad PRISM to obtain the LD50. (B) Balb/c mice, lethally challenged IP with 20 pfu of RVFV, were left untreated or treated IP once a day for 7 days, starting 1 h post-challenge, with JL103 (10 mg/kg). Mice were monitored daily and survival as a Kaplan-Meier plot was compared with the Log-rank (Mantel-Cox) test using GraphPad PRISM to determine the median time-to-death. (PDF) [file ppat.1003297.s012.pdf]
