# Supplementary material for: A Mechanistic Paradigm for Broad-Spectrum Antivirals that Target Virus-Cell Fusion
Source: PLoS Pathog. 2013 Apr 18;9(4):e1003297. doi: 10.1371/journal.ppat.1003297 (PMC3630091; doi:10.1371/journal.ppat.1003297)
Supplement: Figure S7 — Schematic representation of the effect of singlet oxygen (1O2) generated by LJ001 on the phospholipids composing a viral membrane. From top to bottom row: (Fatty acid) Trans-isomerization of linoleic acid after 1O2 attack on C13 following the “ene” reaction. The oxidation results in a hydroperoxide (HpODE) intermediate ultimately reduced into a hydroxyl octadecadienoic (HODE) acid. (Phospholipid) The trans-isomerization of a linoleic acid chain of a 36∶2 phospholipid results in a decreased overall diameter of the phospholipid species and insertion into the highly hydrophobic chain of a polar (less hydrophobic) group. Both the HpODE intermediate and final HODE are represented underneath their corresponding formula drawing. (Membrane) the reduction of the diameter of the 36∶2 phospholipid results in a tighter packing of the phospholipids composing the membrane. Repulsion of the more polar lateral chains also results in a clustering of the oxidized lipids (in microdomains). (Virus) At the scale of the virus, the shrinkage of the particle diameter due to tighter packing of the trans-isomerized unsaturated phospholipids may result in increased positive curvature, while the clustering of the oxidized lipids will result in decreased membrane fluidity. Thus, 1O2-mediated lipid oxidation results in changes in the biophysical properties of the viral membrane that negatively impacts on its ability to undergo virus-cell membrane fusion (see [36], [56]). (PDF) [file ppat.1003297.s007.pdf]

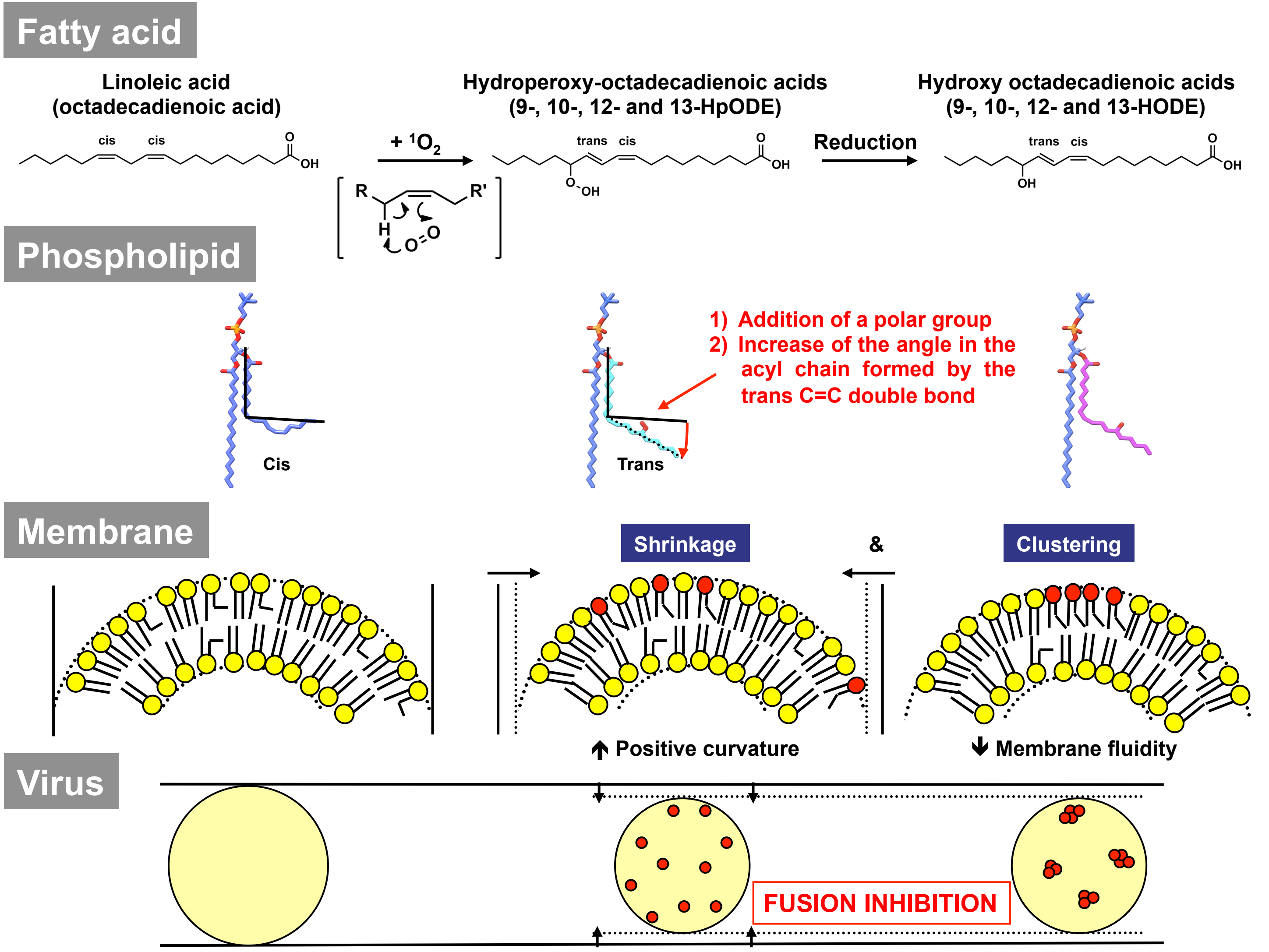

Fatty acid
Linoleic acid
(octadecadienoic acid)
cis
cis
O
OH
+ 1O2
R
R'
H
O=O
Hydroperoxy-octadecadienoic acids
(9-, 10-, 12- and 13-HpODE)
trans
cis
O
OH
Reduction
Hydroxy octadecadienoic acids
(9-, 10-, 12- and 13-HODE)
trans
cis
OH
Phospholipid
Cis
Trans
1) Addition of a polar group
2) Increase of the angle in the acyl chain formed by the trans C=C double bond
Membrane
Shrinkage
&
Clustering
Positive curvature
Membrane fluidity
Virus
FUSION INHIBITION
