# Supplementary material for: A Mechanistic Paradigm for Broad-Spectrum Antivirals that Target Virus-Cell Fusion
Source: PLoS Pathog. 2013 Apr 18;9(4):e1003297. doi: 10.1371/journal.ppat.1003297 (PMC3630091; doi:10.1371/journal.ppat.1003297)
Supplement: Table S1 — Parameters obtained from the fitting of the fluorescence data of partition assays of selected 2-(thio)oxothiazolidin-4-ones (LJ001 and LJ025) and oxazolidine dithiones (JL103, JL118 and JL122). (PDF) [file ppat.1003297.s013.pdf]

Supplementary Table S1. Parameters obtained from the fitting of the fluorescence data of partition assays of selected 2-(thio)oxothiazolidin-4-ones (LJ001 and LJ025) and oxazolidine dithiones (JL103, JL118 and JL122).

	LJ001		*LJ025*		*JL103*		*JL118*		*JL122*	
Lipid Mixture:	K_p x 10^3	I_L / I_w	K_p x 10^3	I_L / I_w	K_p x 10^3	I_L / I_w	K_p x 10^3	I_L / I_w	K_p x 10^3	I_L / I_w
**POPC**	108 ± 33.5	2.29 ± 0.045	23.8 ± 3.01	2.79 ± 0.052	0.58 ± 0.059	14.78 ± 0.652	2.70 ± 0.136	233 ± 3.0	1.13 ± 0.131	143.8 ± 5.4
**HIV-like mixture**	5.19 ± 0.81	2.41 ± 0.095	12.61 ± 3. 52	3.60 ± 0.198	0.31 ± 0.077	68.29 ± 7.624	2.01 ± 0.310	488 ± 18.5	0.267 ± 0.021	613.7 ± 27.8

All measures were made at least in triplicate (see materials and methods), Mean ± SEM is shown. For POPC, a lipid with packing density and fluidity properties similar to mammalian cell membranes, *Kp* values of 108,000 and 23,800 were obtained for LJ001 and LJ025, respectively. When HIV-mimicking membranes were tested, LJ025 exhibited apparently higher *Kp* values than LJ001.

Results indicate that the *Kp* values for the JL compounds (580, 2,700 and 1,130 for JL103, JL118 and JL122, respectively) with POPC are about 1-2 orders of magnitude less than that for LJ001 (108,000) and LJ025 (23,800), but still remain high. The *Kp* values vary less for HIV-mimicking membranes. Despite the lower partitioning of JL compounds into membranes (compared to LJ001), JL103, JL118 and JL122 are much more potent antiviral (IC_{50} < 10-100 fold of LJ001). This indicates that differences in IC_{50}s cannot be due to differential partitioning, and reinforces our statement that the increased antiviral activity of the JL compounds is due to their increased potency as photosensitizers.
