# Supplementary material for: A Mechanistic Paradigm for Broad-Spectrum Antivirals that Target Virus-Cell Fusion
Source: PLoS Pathog. 2013 Apr 18;9(4):e1003297. doi: 10.1371/journal.ppat.1003297 (PMC3630091; doi:10.1371/journal.ppat.1003297)
Supplement: Table S2 — Photophysical properties of selected 2-(thio)oxothiazolidin-4-ones (LJ001 and LJ025) and oxazolidine dithiones (JL102-122). (PDF) [file ppat.1003297.s014.pdf]

Supplementary Table S2 – Photophysical properties of selected 2-(thio)oxothiazolidin-4-ones (LJ001 and LJ025) and oxazolidine dithiones (JL102-122)

Name	Abs Max (nm)	[a]AUC (400-750 nm)	$^1O_2$ QY		[d]k_T × 10^{-7} ($M^{-1}s^{-1}$)
			[b]532 nm	[c]355 nm	
**LJ001**	**455**	**100**	**0**	**0.20 ± 0.02**	**0.189 ± 0.010**
LJ025	405	< 100	NA	0	0.191 ± 0.010
JL102	500	134	0.31 ± 0.03	0.38 ± 0.03	0.607 ± 0.050
**JL103**	**515**	**153**	**0.30 ± 0.03**	**0.36 ± 0.02**	**0.636 ± 0.030**
JL108	530	188	0.28 ± 0.02	0.40 ± 0.04	0.700 ± 0.045
JL109	550	178	0.38 ± 0.02	0.37 ± 0.02	2.000 ± 0.100
**JL118**	**610**	**200**	**0.15 ± 0.02**	**0.14 ± 0.01**	**4.600 ± 0.300**
JL121	380	< 100	NA	0	2.500 ± 0.200
**JL122**	**545**	**153**	**0.23 ± 0.02**	**0.43 ± 0.04**	**1.970 ± 0.050**

[a]Integrated absorption between 400 and 750 nm, divided by the concentration of the compound, and normalized relative to LJ001 (LJ001 = 100). AUC: area under the curve.

[b]Measurements were made in CD_2Cl_2. Reference compound: RB (0.79) at 532 or TPP (0.62) at 532 nm. Average of four measurements, error is one standard deviation. NA: not applicable, these compounds do not absorb at 532 nm.

[c]Measurements were made in CD_2Cl_2. Reference compound C60 (1.00) at 355 nm. Average of four measurements, error is one standard deviation.

[d]Measurements done at 355 nm with C_{60} as external sensitizer. Average of four measurements, error is one standard deviation.

UV-visible spectra integration (AUC). We used UV-visible spectra integrated areas (visible region, 400 nm-750 nm) to estimate the number of photons absorbed by the samples in our antiviral experiments. Typically, solutions of known concentration with maximal absorption of approximately 0.5-1.0 in the visible region were prepared. The integrated absorption areas were obtained via UV-Vis software [Cary WinUV 3.10 (246)], after running each visible spectrum. The Vis integrated absorption ratios were obtained by dividing the integrated absorption area by the concentration of the solution.

Analysis of JL compounds photophysical properties indicated that their absorption spectrum were red-shifted (LJ001 = 455 nm, JL103 = 515 nm, JL122 = 545 nm and JL118 = 610 nm), and that the total integrated absorption (AUC) within the optical spectrum (λ = 400 to 750 nm) was also increasing (JL103 and JL122 = 1.53 times and JL118 = 2 times that of LJ001). Measurements of $^1O_2$ quantum yields (QY) also indicated an increase for the JL compounds: at 355 nm, JL103 and JL122 have a QY about 2 times higher than the one of LJ001, JL118 displays a slightly lower QY. However, whereas LJ001 doesn't absorb light at 532 nm (visible range), hence doesn't generate $^1O_2$ after excitation at this wavelength, all JL compounds have a high $^1O_2$ QY at 532 nm. These results confirm that all JL compounds are more efficient in generating $^1O_2$ than LJ001. Of note, all these photosensitizers have relatively small rates of $^1O_2$ removal (k_T) indicating that self-quenching of $^1O_2$ by the photosensitizer-drugs (*i.e.* oxidation of the photosensitizers themselves by the $^1O_2$ they generate) is minor and is not significantly limiting their antiviral function.
