# Supplementary material for: A Mechanistic Paradigm for Broad-Spectrum Antivirals that Target Virus-Cell Fusion
Source: PLoS Pathog. 2013 Apr 18;9(4):e1003297. doi: 10.1371/journal.ppat.1003297 (PMC3630091; doi:10.1371/journal.ppat.1003297)
Supplement: Table S3 — Antiviral activity, cytotoxicity, and therapeutic indexes of selected 2-(thio)oxothiazolidin-4-ones (LJ001 and LJ025) and oxazolidine dithiones (JL101-JL122). (PDF) [file ppat.1003297.s015.pdf]

Supplementary Table S3 – Antiviral activity, cytotoxicity, and therapeutic indexes of selected 2-(thio)oxothiazolidin-4-ones (LJ001 and LJ025) and oxazolidine dithiones (JL101-JL122)

Name	[(a)] IC_{50} ± SD (nM) (N=x)			[(b)] CC_{50} (nM)	[(c)] TI (NDV)
	HIV	HSV	NDV	PBMC	
**LJ001**	**181.9 ± 244.4 (11)**	**35.3 ± 48.3 (3)**	**180.6 ± 104.7 (13)**	**11,940**	**66**
LJ025	NA	NA	NA	> 50,000	-
JL101	-	-	8,800 ± 8,900 (3)	-	-
JL102	60.0 ± 63.1 (5)	5.1 ± 4.8 (4)	31.8 ± 16.9 (7)	14,780	465
**JL103**	**12.2 ± 9.7 (5)**	**1.9 ± 2.5 (5)**	**5.7 ± 6.4 (14)**	**9,140**	**1,604**
JL108	10.8 ± 4.5 (2)	-	4.8 ± 3.0 (5)	7,050	1,469
JL109	4.5 ± 4.8 (3)	0,7 ± 0,6 (3)	3.8 ± 3.9 (6)	8,510	2,239
JL111	22.0 ± 18.4 (2)	6.0 (1)	9.4 ± 4.2 (4)	-	-
JL117	-	3.2 ± 2.5 (2)	4.3 ± 3.0 (2)	-	-
**JL118**	**11.7 ± 5.5 (3)**	**10.1 ± 8.6 (3)**	**2.1 ± 1.4 (6)**	**37,050**	**17,643**
JL121	NA	NA	NA	> 50,000	-
**JL122**	**64.0 ± 41.5 (3)**	**2.4 ± 1.4 (5)**	**0.9 ± 0.8 (2)**	**10,280**	**11,422**

[(a)] IC_{50} measured after 10 min of light exposure of the treated viruses. N=x: Number of independent repeats.
[(b)] Concentration of compound inducing 50% of cytotoxicity, as measured by MTT assay, after 10 min of light exposure of the treated cells.
[(c)] Therapeutic index calculated as CC_{50}/IC_{50} against NDV.
NA: Not active.
-: Not determined.

Cytotoxicity assay. Freshly isolated peripheral blood mononuclear cells (PBMC) were seeded into a 96-wells plate (50,000 cells/well) and incubated for 24h in complete medium (RPMI, 20% FBS, 10 U/ml Il-2, 1% Pen/Strep) before treatment with the indicated compounds. Serial dilutions of the compounds were added to the cells, and the plates were exposed to light in the same conditions as viruses (10 min at room temperature) before returning to the incubator wrapped in foil. The following day remaining cell viability was measured by MTT assay (Vybrant MTT, Invitrogen). Results were expressed as the percentage of untreated cells (100% survival) and graphed against the Log_{10} of the concentration using GraphPad PRISM™. Non-linear regression was used to determine the concentrations corresponding to a 50% decrease in cell viability (cytotoxicity 50, CC_{50}).
