# Supplementary material for: A Mechanistic Paradigm for Broad-Spectrum Antivirals that Target Virus-Cell Fusion
Source: PLoS Pathog. 2013 Apr 18;9(4):e1003297. doi: 10.1371/journal.ppat.1003297 (PMC3630091; doi:10.1371/journal.ppat.1003297)
Supplement: Table S4 — Pharmacokinetics of selected 2-(thio)oxothiazolidin-4-one (LJ001) and oxazolidine dithiones (JL103, JL118, JL122). (PDF) [file ppat.1003297.s016.pdf]

Supplementary Table S4 – Pharmacokinetics of selected 2-(thio)oxothiazolidin-4-one (LJ001) and oxazolidine dithiones (JL103, JL118, JL122)

Drug	Dose Route	Dose Level (mg/kg)	C_P or C_{max}[a] (ng/ml)	T_{max} (hr)	AUC_{last} (hr•ng/ml)	AUC_{inf} (hr•ng/ml)	MRT_{last} (hr)	$t_{1/2}$ (hr)	F (%)	V[e] (L/kg)	Cl[e] (ml/hr/kg)
LJ-001	iv	10	767.0	0.08	238	241	0.2	0.2	NA[c]	9.1	41549
	ip	5	56.2	0.08	15	17	0.2	0.2	14.1	9.1	41825
	po	10	7.9[b]	0.5	NC	NC[d]	NC	NC	NC	NC	NC
	po	100	5.4[b]	0.08	NC	NC	NC	NC	NC	NC	NC
JL-103	iv	10	16167.0	0.08	6464	6498	1.1	2.2	NA	4.8	1539
	ip	10	612.7	0.5	2894	2959	4.2	4.1	45.5	9.1	1538
	po	10	1126.7	1	3093	3115	2.3	1.6	47.9	3.6	1538
	po	100	417.0	1	1947	1986	3.6	2.2	3.1	4.9	1561

[a] C_P is the first measured plasma concentration for iv groups.

[b] There was only one sample with detectable drug levels.

[c] NA = not applicable.

[d] NC = not calculated. There were insufficient data points for calculation of parameter.

[e] For ip and po groups, V and Cl were calculated from F•(V/F) and F•(Cl/F), respectively.

Test Compound	Route	Dose (mg/kg)	C_{max} (ng/ml)	T_{max} (hr)	AUC_{last} (hr*ng/ml)	AUC_{inf} (hr*ng/ml)	MRT_{last} (hr)	$t_{1/2}$ (hr)	V/F (L/kg)	Cl/F (ml/hr/kg)
JL-118	ip	1.25	626	0.5	1835	2247.92	5.3	16.0	12.8	556.1
JL-118	po	10	253	1	1347.67	*NC*[a]	8.8	*NC*[a]	*NC*[a]	*NC*[a]
	po	50	122	0.5	841.24	*NC*[a]	9.8	*NC*[a]	*NC*[a]	*NC*[a]
	po	100	124	0.5	1159.83	*NC*[a]	11.4	*NC*[a]	*NC*[a]	*NC*[a]
JL-122	ip	5	122	0.5	568.61	*NC*[a]	5.5	*NC*[a]	*NC*[a]	*NC*[a]
	ip	10	132	0.25	924.18	*NC*[a]	6.2	*NC*[a]	*NC*[a]	*NC*[a]
JL-122	po	10	99.3	0.5	231.79	*NC*[a]	4.7	*NC*[a]	*NC*[a]	*NC*[a]
	po	100	287	1	1271.74	*NC*[a]	7.0	*NC*[a]	*NC*[a]	*NC*[a]

[a] NC - Not calculated, due to limited (≤ 2) number of time-points with measurable plasma concentrations in the terminal elimination phase after T_{max} or a poor fit ($r^2 \leq 0.8$) for the straight line portion in the terminal elimination phase

iv: intravenous. ip: intraperitoneal. po: per os (oral). C_p: plasma concentration. C_{max}: observed maximum plasma concentration after administration. T_{max}: time to reach C_{max}. AUC_{last}: area under the concentration-time curve up to the last measurable concentration. AUC_{inf}: AUC curve to infinite time. MRT_{last}: mean residence time of the drug in the systemic circulation from 0 to last time point. $t_{1/2}$: terminal half-life. F: absolute bioavailability. V: volume of distribution. Cl: total plasma serum or blood clearance of drug.
