# Supplementary material for: A Mechanistic Paradigm for Broad-Spectrum Antivirals that Target Virus-Cell Fusion
Source: PLoS Pathog. 2013 Apr 18;9(4):e1003297. doi: 10.1371/journal.ppat.1003297 (PMC3630091; doi:10.1371/journal.ppat.1003297)
Supplement: Text S1 — Supporting information. Supporting Materials and Methods. (DOC) [file ppat.1003297.s017.doc]

**SUPPORTING MATERIALS AND METHODS**

**CHEMISTRY**

**Solvents, reagents and synthetic procedures**

All reactions were carried out under an argon atmosphere unless otherwise specified. Toluene, and benzene were distilled from benzoquinone ketyl radical under an argon atmosphere. Dichloromethane (DCM), triethylamine (TEA) were distilled from calcium hydride under an argon atmosphere. 3-Ethyl-2-thioxooxazolidin-4-one, 5-bromofurfural and 5-phenyl-2- were purified according to literature procedures. ^1^H NMR spectra were recorded on Bruker spectrometers (at 400 MHz) and are reported relative to deuterated solvent signals. Data for ^1^H NMR spectra are reported as follows: chemical shift (δ ppm), multiplicity, coupling constant (Hz) and integration. Splitting patterns are designated as follows: s, singlet; d, doublet; t, triplet; q, quartet; m, multiplet; and br, broad. ^13^C NMR spectra were recorded on Bruker Spectrometers (at 100 MHz). Data for ^13^C NMR spectra are reported in terms of chemical shift. The chemical shifts are reported in parts per million (ppm, δ). The reactions were monitored with a silica gel TLC plate under UV light (254 nm) followed by visualization with a *p*-anisaldehyde or phosphomolybdic acid staining solution. Column chromatography was performed on silica gel 60, 70-230 mesh. HRMS (ESI) spectra were recorded on Waters LCT premier with ACQUITY LC spectrometer.

**Synthesis of the compounds**

We were able to prepare a series of oxazolidine-2,4-dithiones by a straightforward synthesis. Reaction of the commercially available 3-ethyl-2-thiooxazolidine-4-one (1), which could also be prepared by the reaction of methyl glycolate with ethyl isothiocyanate, with Lawesson’s reagent afforded the dithione (2) in 87% yield. A series of aldehydes were prepared by Suzuki coupling of 5-bromo-2-furaldehyde (3) with various arylboronic acids to give (4) in yields of 24-80%. The final condensation of the oxazolidine-2,4-dithione (2) and the aldehydes (4) gave a series of analogues (5), which we have named the **JL** series. In addition, we condensed (1) with the aldehyde (Ar = Ph) to give the oxo analogue JL101. The overall synthetic scheme to JL series is shown below.

**Representative procedure for JL series from 3-ethyl-2-thioxooxazolidin-4-one (1) and 5-bromofurfural (3) via thionylation, Suzuki coupling and Knovenagel condensation. (JL103)**

**Thionylation reaction for 3-Ethyloxazolidine-2,4-dithione (2).**

A toluene (10 mL) solution of 3-ethyl-2-thioxooxazolidin-4-one (1) (145 mg, 1.0 mmol) and Lawesson’s reagent (607 mg, 1.5 mmol) was refluxed overnight. The solution was cooled and diluted with ethyl acetate (100 mL), then washed with water (2 X 20 mL). The organic layer was dried with brine and MgSO_4_, and concentrated *in vacuo*. The residue was purified by flash column chromatography on silica gel (hexane:ethyl acetate=10:1), and the desired product (2) was obtained in 94% yield (152 mg, orange oil). ^1^H NMR (400 MHz, CDCl_3_): δ 5.12 (s, 2H), 4.21 (q, *J* = 7.2 Hz, 2H), 1.29 (t, *J* = 7.2 Hz, 3H).

**Suzuki coupling reaction for 5-(2-Methoxyphenyl)-2-furaldehyde (4)**

To a benzene (10 mL) solution of 5-bromo-2-furaldehyde (3) (350 mg, 2.0 mmol) and 2-methoxyphenyl-boronic acid (304 mg, 2.0 mmol) was added tetrakis(triphenyl-phosphine)palladium(0) (69.4 mg, 0.06 mmol) and *sat. aq.* K_2_CO_3_ solution (2.0 ml) at 21 °C under an argon atmosphere. The reaction mixture was refluxed for 2 h. The mixture was then cooled and diluted with ethyl acetate (100 mL). The organic layer was washed with water (2 X 20 mL), then dried with brine and MgSO_4_ and concentrated *in vacuo*. Flash column chromatography on silica gel (hexane:ethyl acetate = 20:1) of the residue afforded 5-(2-methoxyphenyl)-2-furaldehyde (4) in 65% yield (265 mg, yellow oil). ^1^H NMR (400 MHz, CDCl_3_): δ 9.65 (s, 1H), 8.05 (dd, *J* = 8.0, 2.0 Hz, 1H), 7.39-7.35 (m, 1H), 7.33 (d, *J* = 3.6 Hz, 1H), 7.14 (d, *J* = 4.0 Hz, 1H), 7.07 (dt, *J* = 7.2, 0.8 Hz, 1H), 7.00 (bd, *J* = 8.0 Hz, 1H), 3.97 (s, 3H).

**Knovenagel condensation reaction for (*Z*) 3-Ethyl-5-[5-(2-methoxyphenyl)-furan-2-ylmethylene]oxazolid-ine-2,4-dithione (5, JL103).**

To a mixture of 3-ethyloxazolidine-2,4-dithione (2) (79 mg, 0.49 mmol), 5-(2-methoxyphenyl)-2-furaldehyde (4) (99 mg, 0.49 mmol) and sodium acetate (201 mg, 2.45 mmol) was added acetic acid (5 mL). As more of the desired product was formed, the reaction mixture color turned to a very red solution. The reaction mixture was refluxed overnight, then the excess solvent was removed *in vacuo*. The residue was diluted with ethyl acetate (100 mL), then washed with water (2 X 20 mL). The organic layer was dried with brine and MgSO_4_, then concentrated *in vacuo*. Flash column chromatography afforded the desired product (5, JL103) as a dark purple solid (160 mg, 95%): mp 168-169 ^o^C; ^1^H NMR (400 MHz, CDCl_3_): δ 7.98 (dd, *J* = 8.0, 1.6 Hz, 1H), 7.37-7.33 (m, 2H), 7.19 (d, *J* = 4.0 Hz, 1H), 7.08 (t, *J* = 3.6 Hz, 1H), 7.05 (s, 1H), 6.98 (bd, *J* = 8.4 Hz, 1H), 4.30 (q, *J* = 7.2 Hz, 2H), 3.97 (s, 3H), 1.34 (t, *J* = 7.2 Hz, 3H); ^13^C NMR (100 MHz, CDCl_3_): δ 182.6, 182.3, 156.5, 155.5, 147.5, 146.8, 130.2, 127.0, 122.9, 121.1, 118.3, 114.9, 111.2, 103.3, 55.5, 41.6, 11.3 ppm; IR (NaCl) 2967, 2921, 2845, 1622, 1505, 1486, 1391, 1351, 1304, 1271, 1245, 1114, 1078, 1022, 952, 788, 752 cm^-1^; HRMS calculated for C_17_H_15_NO_3_S_2_: 345.0532; Found: 346.0544 [M+H]^+^.

**Characterization of selected LJ and JL compounds**

(Z)-3-allyl-5-((5-phenylfuran-2-yl)methylene)-2-thioxothiazolidin-4-one (**LJ001**)

Orange solid: mp 127-128 ^o^C; ^1^H NMR (400 MHz, CDCl_3_): δ 7.78 (dd, *J* = 8.4, 1.2 Hz, 2H), 7.50-7.46 (m, 2H), 7.47 (s, 1H), 7.41-7.36 (m, 1H), 6.94 (d, *J* = 3.6 Hz, 1H), 6.85 (d, *J* = 3.6 Hz, 1H), 5.93-5.84 (m, 1H), 5.33-5.24 (m, 2H), 4.75 (d, *J* = 5.6 Hz, 2H); ^13^C NMR (100 MHz, CDCl_3_): δ 194.1, 167.2, 159.0, 149.4, 129.7, 129.3, 129.1, 128.9, 124.7, 121.5, 119.8, 119.2, 118.1, 108.9, 46.4 ppm; HRMS (ESI^+^) calculated for C_17_H_13_NO_2_S_2_: 327.0466; Found: 328.0457 [M+H]^+^.

(Z)-3-allyl-5-((5-phenylfuran-2-yl)methylene)thiazolidine-2,4-dione (**LJ025**)

Yellow solid: mp 149-151 ^o^C; ^1^H NMR (400 MHz, CDCl_3_): δ 7.75 (dd, *J* = 8.4, 1.2 Hz, 2H), 7.64 (s, 1H), 7.48-7.44 (m, 2H), 7.39-7.34 (m, 1H), 6.88 (d, *J* = 3.6 Hz, 1H), 6.83 (d, *J* = 3.6 Hz, 1H), 5.92 (m, 1H), 5.32-5.23 (m, 2H), 4.35 (d, *J* = 6.0 Hz, 2H); ^13^C NMR (100 MHz, CDCl_3_): δ 168.6, 165.7, 158.1, 149.0, 130.4, 129.1, 129.1, 124.6, 124.6, 120.4, 119.2, 118.7, 118.3, 108.5, 43.7 ppm; HRMS (ESI^+^) calculated for C_17_H_13_NO_3_S: 311.0693; Found: 312.0687 [M+H]^+^.

(Z)-5-((5-(2,4-dimethoxyphenyl)furan-2-yl)methylene)-3-ethyloxazolidine-2,4-dithione (**JL109**)

Dark purple solid: mp 227-228 ^o^C; ^1^H NMR (400 MHz, CDCl_3_): δ 8.92 (d, *J* = 4.0 Hz, 1H), 7.36 (d, *J* = 3.6 Hz, 1H), 7.08 (d, *J* = 3.6 Hz, 1H), 7.06 (s, 1H), 6.64 (dd, *J* = 8.8, 2.4 Hz, 1H), 6.53 (d, *J* = 2.4 Hz, 1H), 4.30 (q, *J* = 7.2 Hz, 2H), 4.05 (s, 3H), 3.95 (s, 3H), 1.34 (t, *J* = 7.2 Hz, 3H); ^13^C NMR (100 MHz, CDCl_3_): δ 182.4, 181.1, 162.0, 157.9, 147.2, 146.5, 129.2, 128.3, 123.6, 120.6, 113.6, 111.8, 105.6, 103.7, 98.9, 55.5, 41.8, 11.2 ppm; HRMS (ESI^+^) calculated for C_18_H_17_NO_4_S_2_: 375.0677; Found: 376.0667 [M+H]^+^.

(Z)-5-((5-(4-(dimethylamino)phenyl)furan-2-yl)methylene)-3-ethyloxazolidine-2,4-dithione (**JL118**)

Dark green solid: mp 199-201 ^o^C; ^1^H NMR (400 MHz, CDCl_3_): δ 7.67 (d, *J* = 8.8 Hz, 2H), 7.37 (bd, *J* = 4.0 Hz, 1H), 7.05 (s, 1H), 6.74 (d, *J* = 8.8 Hz, 2H), 6.73 (d, *J* = 4.4 Hz, 1H), 4.29 (q, *J* = 7.2 Hz, 2H), 3.04 (s, 6H), 1.34 (t, *J* = 7.2 Hz, 3H); ^13^C NMR (100 MHz, CDCl_3_): δ 182.1, 181.6, 160.9, 151.2, 147.7, 146.2, 126.5, 124.3, 117.1, 112.1, 107.9, 103.8, 41.5, 40.2, 11.3 ppm; HRMS (ESI^+^) calculated for C_18_H_18_N_2_O_2_S_2_: 358.0888; Found: 359.0873 [M+H]^+^.

(Z)-3-ethyl-5-((5-(2,4,6-trimethoxyphenyl)furan-2-yl)methylene)oxazolidine-2,4-dithione (**JL122**)

Black solid: mp 166-167 ^o^C; ^1^H NMR (400 MHz, CDCl_3_): δ 7.35 (bd, *J* = 3.6 Hz, 1H), 7.07 (s, 1H), 6.90 (d, *J* = 4.0 Hz, 1H), 6.20 (s, 2H), 4.30 (q, *J* = 7.2 Hz, 2H), 3.90 (s, 6H), 3.87 (s, 3H), 1.34 (t, *J* = 7.2 Hz, 3H); ^13^C NMR (100 MHz, CDCl_3_): δ 182.4, 182.3, 162.5, 159.8, 155.1, 147.3, 146.4, 122.9, 115.9, 104.2, 101.7, 91.2, 56.3, 55.5, 41.6, 11.3 ppm; HRMS (ESI^+^) calculated for C_19_H_19_NO_5_S_2_: 405.0783; Found: 406.0779 [M+H]^+^.

**BIOLOGY AND PHYSICAL MEASUREMENTS**

**UV-visible spectra integration (AUC)**. We used UV-visible spectra integrated areas (visible region, 400 nm-750 nm) to estimate the number of photons absorbed by the samples in our antiviral experiments. Typically, solutions of known concentration with maximal absorption of approximately 0.5-1.0 in the visible region were prepared. The integrated absorption areas were obtained via UV-Vis software [Cary WinUV 3.10 (246)], after running each visible spectrum. The Vis integrated absorption ratios were obtained by dividing the integrated absorption area by the concentration of the solution.

**Viral Strains.** We used the following viral strains: Vesicular stomatitis virus (VSV)-RFP; Ebola Zaire (EBOV); Rift Valley fever (RVFV) ZH501 (*in vivo* challenge) and MP-12 (vaccine strain, *in vitro* IC_50_); human immunodeficiency virus (HIV)-1 JRCSF (R5-tropic) or IIIB (X4-tropic); Semliki forest virus (SFV); Cytomegalovirus (CMV, strain T3259); Rabies virus (RABV, Challenge Virus Standard); Adenovirus (Ad)-5-eGFP; Influenza A A/PR/8/34 (H1N1); Nipah virus (NiV) Malaysia; Hendra virus (HeV); Herpes simplex virus (HSV)-1-GFP and Newcastle disease virus rNDV/F3aa-GFP (NDV).

The use of the various agents listed herein was approved by the various *ad hoc* biosafety committees. VSV, RVFV-MP12, CMV, Ad5, H1N1, HSV-1 and NDV were handled under BSL2 conditions. Note that the strain of NDV used is lentogenic and thus is not regulated as select agent. SFV and HIV were handled under BSL2-enhanced conditions (BSL2+). RABV was handled as ACDP3/SAPO4 in custom facilities. EBOV, RVFV-ZH501, NiV and HeV were handled under BSL4 conditions.

**Determination of the IC_50_.** Viruses were treated at multiplicities of infection (MOIs) within the linear range of infection (0.2-0.4). In general, after serial dilution of the compounds, in clear transparent eppendorf tubes, an equal volume of virus (2X) was added, and mixed. To maximize light-exposure, the tubes were subsequently laid on their side and exposed for 10 minutes to the white fluorescent light source of the biosafety cabinet(s) (BSC) lamps. BSCs’ light source lamp consists of two fluorescent tubes of 85W each. The plates were on average 75-100 cm away from the light source. The treated suspensions were then used to infect cells from which the culture medium was just removed. After 1-2h in an incubator at 37°C, the inoculum was completely removed and replaced with fresh culture medium containing no drug. No additional wash was performed. The plates were kept away from light until the end point of the assay. Experiments in the dark were carried out in a dark room using a red safelight to illuminate the BSC. Infection was determined by luciferase activity in cell lysate (HIV), fluorescence-activated cell sorting (FACS) (VSV-RFP, HSV-GFP, NDV-GFP and Ad5-GFP), plaque assay (EBOV, RVFV MP-12, H1N1, NiV and HeV), immunofluorescence (SFV) or secreted embryonic alkaline phosphatase (SEAP) activity (CMV) and expressed relative to untreated virus (100%). Values were graphed and IC_50_ calculated by non-linear regression curve fitting with variable slopes constraining the top to 100% and the bottom to 0%, using GraphPad PRISM software.

Despite the multicentric character of this study (and light sources in BSCs), comparable IC_50_ values and trends were obtained against various representative viruses.

**Cytotoxicity assay.**  Freshly isolated peripheral blood mononuclear cells (PBMC) were seeded into a 96-wells plate (50,000 cells/well) and incubated for 24h in complete medium (RPMI, 20% FBS, 10 U/ml Il-2, 1% Pen/Strep) before treatment with the indicated compounds. Serial dilutions of the compounds were added to the cells, and the plates were exposed to light in the same conditions as viruses (10 min at room temperature) before returning to the incubator wrapped in foil. The following day remaining cell viability was measured by MTT assay (Vybrant MTT, Invitrogen). Results were expressed as the percentage of untreated cells (100% survival) and graphed against the Log_10_ of the concentration using GraphPad PRISM. Non-linear regression was used to determine the concentrations corresponding to a 50% decrease in cell viability (cytotoxicity 50, CC_50_).

**Virus inactivation in red blood cells.** Packed red blood cells (RBC) were obtained from unused, processed, and pre-screened donor units from the UCLA Blood Bank. All donor units were negative for HIV and other pathogens as dictated by AABB standards. Stock solutions of HIV-1_JR-CSF_ (1:10 final volume, ~ 100 μg p24 equivalents) were spiked into packed RBC resuspended in PBS, and then diluted with additional PBS to reach the desired hematocrit (hct) (volume % RBC). Subsequently, 500 μl aliquots were distributed in the wells of a 12 wells plate, so that the resulting thickness of the layer was about 1 mm. Compounds were added to the wells (50 μl, 20 μM final), and the plates were illuminated, under continuous agitation (100 rpm) for 1h. The “light-exposure” control consisted of wells treated with the vehicle (DMSO) only, and the “dark” control consisted of plate wrapped in foil, for the duration of the experiment, after addition of the compounds to avoid light-activation. No antiviral effect was observed in either of these controls. Samples were subsequently centrifuged (12,000 rpm, 1 min) and the supernatant used to infect reporter TZM-bl cells to evaluate the amount of remaining infectious HIV. Infection was measured as mentioned above by luciferase assay in cell lysates. Results are reported as % inhibition relative to the “light-exposure” control.

**Reagents for membrane biophysical assays.** POPC (1-palmitoyl-2-oleyl-*sn*-glycero-3-phosphocholine), DPPC (1,2-dipalmitoyl-*sn*-glycero-3-phosphocholine), POPE (1-palmitoyl-2-oleoyl-*sn*-glycero-3-phosphoethanolamine) and POPS (1-palmitoyl-2-oleoyl-sn-glycero-3-phospho-L-serine) were purchased from Avanti Polar Lipids (Alabaster, AL, USA). Cholesterol, SM (sphingomyelin), DPH (1,6-diphenyl-1,3,5-hexatriene) and TMA-DPH (4’-(trimethylammonio)diphenylhexatriene p-toluenesulfonate) were from Sigma (St. Louis, MO, USA).

The working buffer used throughout partition, surface pressure and anisotropy studies was HEPES 10 mM pH 7.4 in NaCl 150 mM. Large unilamellar vesicles (LUV) were prepared by extrusion methods, as described elsewhere .

For the PBMC, human blood samples were obtained from healthy volunteers with their written informed consent at the Instituto Português do Sangue (Lisboa, Portugal), as approved by the ethics committee of the Faculdade de Medicina da Universidade de Lisboa. Peripheral blood mononuclear cells (PBMC) were isolated by density gradient using Ficoll-Paque Plus (GE Healthcare, Little Chalfont, UK) and counted in a Neubauer improved hemocytometer.

**Lipid oxidation (liposomes) experiment.** In order to determine if LJ001 and JL103 were capable of oxidizing the fatty acids present in membrane phospholipids, both active compounds were combined with recombinant liposomes; the experimental groups were exposed to light; and the amounts of select oxidized fatty acids present in the groups were determined by LC-MS/MS. First, for each group 150 µg of recombinant liposomes, whose fatty acid content was over 60% linoleic acid, was brought up to 1 ml in PBS. Second, a liposome only control; liposome plus 10 µM of the inactive compound LJ025; liposome plus 10 µM of the active compound LJ001; and liposome plus 10 µM of the active compound JL103 were exposed to the light of a BSC for 1 hour. The fatty acids were then de-esterified, the lipids were isolated by solid-phase extraction, and the samples were prepared for LC-MS/MS analysis as described below. The lipid extracts were then analyzed for the presence of two oxidized products of linoleic acid, 13HODE and 9HODE, also as described below.

**Lipid extraction and sample preparation for LC/MS/MS analysis.** 13HETE-d_4_ (Cayman, 338610)) was added to each sample as an internal standard (100 µL of 100 ng 13HODE-d_4_/ml methanol). 50 µM BHT was also added, in order to prevent further oxidation of the samples. The fatty acids of each sample were then de-esterified by adding equal volume 1 M KOH and incubating for thirty minutes at 37°C. Enough 1 M HCl was then added to bring the final pH to between 3 and 4. The samples were spun down at 13K rpm for 10 min at 4°C to pellet any precipitates. The supernatants were then removed for further lipid extraction. Oasis HLB 3 cc (60mg) solid-phase extraction cartridges (Waters, 186001880) on a vacuum manifold were equilibrated with 2 ml methanol followed by 2 ml water. The sample supernatants were slowly loaded onto the cartridges under vacuum. After the samples had completely flowed through at approximately 0.5 ml/min, the cartridges were washed with 2 ml 5% methanol in water. Lipids were then eluted from the cartridges with 2 ml methanol. The eluates were evaporated to dryness under argon at 37° C. Each dried lipid extract was then re-suspended in 100 µl of methanol using gentle vortexing. The samples were transferred to autosampler vials (Fisher scientific, 03-396-74) for LC-MS/MS analysis.

**LC-MS/MS analysis.** LC-MS/MS was performed using a 4000 QTRAP quadruple mass spectrometer (Applied Biosystems) equipped with electrospray ionization source. The HPLC system utilized an Agilent 1200 series LC pump equipped with a thermostatted autosampler (Agilent Technologies). Chromatography was performed using a Luna C-18(2) column (3 µm particle, 150 × 3.0mm; Phenomenex) with a security guard cartridge (C-18; Phenomenex) at 40^o^C. Mobile phase A consisted of 0.1% formic acid in water, and mobile phase B consisted of 0.1% formic acid in acetonitrile. The autosampler was set at 4^o^C. The injection volume was 10 µl, and the flow rate was controlled at 0.4 ml/min. The gradient program was as follows: 0-2 min, 50% B; 2-3 min, linear gradient from 50% to 60% B; 3-15 min, linear gradient from 60-65% B; 15-17 min, 65% B; 17-19 min, linear gradient from 65-100% B; 19-21 min 100% B; 21-23 min, linear gradient from 100% to 50% B; 23-27 min, 50% B. The data acquisitions and instrument control were accomplished using Analyst 1.4.2 software (Applied Biosystems). Detection was accomplished by using the multiple reaction monitoring (MRM) mode with negative ion detection; the parameter settings used were: ion spray voltage=-4500 V; curtain gas=20 (nitrogen); ion source gas 1=50; ion source gas 2=30; ion source gas 2 temperature=550^o^C. Collision energy, declustering potential and collision cell exit potential were optimized for each compound to obtain optimum sensitivity. The transitions monitored were mass-to-charge ratio (m/z): m/z 295 🡪 194.8 for 13HODE; 295 🡪 171 for 9HODE; and 299 🡪 197.9 for 13HODE-d_4_.

**Influenza lipidome analysis.** Influenza A virus (A/PR/8/34 H1N1) was treated with 5 μM of LJ001 or the negative control LJ025, exposed to light for 1 h, and subsequently subjected to lipid extraction. Analyses of lipids, including oxidized species, were carried out using a high-resolution Thermo LTQ-Orbitap mass spectrometer and an ABI 3200 QTRAP mass spectrometer after liquid chromatography separation .

***In vivo* challenge**. All animal studies were approved by the Institutional Animal Care and Use Committee (IACUC) at the University of Texas Medical Branch (UTMB) and performed at the Robert E. Shope biosafety level 4 (BSL-4) laboratory. Balb/c mice were challenged with Rift Valley fever virus (RVFV ZH501 strain) via the intraperitoneal (ip) route. Treatments of mice with JL103 (10 mg/kg), JL118 (1.25 mg/kg), and JL122 (10 mg/kg) were performed at the indicated times post infection (ip injections). Compound concentrations were based on results from previously performed pharmacokinetic studies. The vehicle used was 15% DMSO/85% PEG400. The dosing regimens corresponding to each experiment are indicated in figures legends.

**Pharmacokinetics (PK) profiles of LJ001, JL102 and JL103 (SRI Study No. B435-10) and JL109, JL118 and JL122 (SRI Study No. B492-1l).**

The pharmacokinetic parameters of each test compound are presented in table S2.

SRI International performed these studies. The objective was to determine the pharmacokinetic (PK) profile of LJ001, JL102, JL103, JL109, JL118 and JL122 after a single oral gavage (po), intraperitoneal (ip) or intravenous (iv) administration to female Balb/c mice. The purpose of this study was to provide data that can be used to support research efforts. It was exploratory and not within the scope of U.S. Food and Drug Administration (FDA) “Good Laboratory Practice for Nonclinical Laboratory Studies” (GLP) regulations, as described in 21 CFR Part 58. Nevertheless, the study was planned, performed, recorded, and reported in accordance with standard practices to ensure data quality and integrity. General procedures for animal care and housing were in accordance with the National Research Council (NRC) *Guide for the Care and Use of Laboratory Animals* (1996) and the Animal Welfare Standards incorporated in 9 CFR Part 3, 1991.

**Mice.** Female Balb/c (Charles River (Hollister, CA)), 7-8 weeks, 15-25 g at injection.

**Formulation** (all dose administration routes). LJ001, JL109, JL118, and JL122: 15% DMSO/85% PEG 400

JL102 and JL103: 15% Solutol/15% PEG 400/70% sterile water

The LJ001, JL109, JL118 and JL122 dose formulations were prepared by dissolving the appropriate amount of LJ001 in DMSO, mixing the formulations with a sonicator and vortex for 8-30 min. The appropriate amount of PEG 400 was then added and the formulations were mixed with a vortex. The JL102 and JL103 dose formulations were prepared by adding melted Solutol to the appropriate amount of test article and mixing the formulations with a vortex and sonicator containing hot water (~45°C) for 10-30 min. The appropriate amount of PEG 400 was added and the formulations were mixed with a vortex for 1 min. Sterile water was then added and the formulations were then mixed with a vortex for an additional 1 min. Dosing formulations for iv and ip administration were filter sterilized.

**Dose administration.** Mice were treated with a single dose of LJ001, JL102 or JL103 at 10 mg/kg iv, ip and po, and 100 mg/kg po. In the 3 iv dose groups, clinical signs, including tremors, hypoactivity or rough fur, were observed in some of the mice. Out of the first 3 mice treated with LJ-001 at 10 mg/kg ip, 2 were sacrificed in moribund condition and 1 was found dead shortly after dose administration. These mice were replaced and the dose level was reduced to 5 mg/kg. Mice treated with LJ001 at 5 mg/kg ip, and JL102 and JL103 at 10 mg/kg ip appeared normal. Hypoactivity was the only clinical observation noted in some of the mice treated with the 3 test articles at 10 and 100 mg/kg po. Dose reductions were also made in JL-109 and JL-118 ip groups from 2.5 mg/kg to 1.25 mg/kg as deaths occurred in the initial 3 animals in each group at about 15 min post administration at the 2.5 mg/kg dose level. These animals were replaced and administered with the lower ip dose level of 1.25 mg/kg and underwent blood collections. Mice received single ip doses of 1.25 mg/kg (JL109 and JL118) or 5 mg/kg (JL122) and po doses of 10 mg/kg (JL109, JL118, JL122), 50 mg/kg (JL109, JL118), or 100 mg/kg (JL109, JL118, JL122). Most animals appeared normal with a few exceptions with ruffled fur and hunched posture.

**Plasma drug levels.** Blood was collected from the retro-orbital sinus of mice under isoflurane anesthesia into tubes containing K_3_EDTA, processed to plasma, and then stored frozen at ≤-70°C). For each group, blood was collected from 3 mice per time point at 5, 15, 30 and 60 min, and 3, 6, 12 and 24 hr post-dose. Each mouse was used for only 2 time points with the second collection time point being terminal. Blood was collected from 5 untreated mice for baseline control samples. Drug levels were determined in collected plasma samples by LC-MS/MS using a bioanalytical method developed at SRI. The effects of light on compound stability/purity were also included.

The plasma drug level data were analyzed using WinNonlin® version 5.2 Professional by noncompartmental modeling. The dose administered was input to the program as mg/kg, and as a result no additional corrections for individual body weights of the animals were necessary.

The following parameters and constants were determined: first measured plasma concentration after iv administration (CP), maximum plasma concentration after ip and po administration (Cmax), time to maximum plasma concentration (Tmax), area under the plasma concentration-time curve (AUC), volume of distribution (V), elimination clearance (Cl), mean residence time to the last time point (MRTlast) and terminal elimination half-life (t1/2). Bioavailability (F) of the extravascular dose routes (ED) was calculated as: (AUCinf ED / AUCinf iv) • (Doseiv / DoseED) • 100.

**Pharmacokinetics analysis.** Plasma levels in the iv (10 mg/kg) and ip (5 mg/kg) groups for LJ001 declined quickly after administration and were detectable up to 1 and 0.5 hr post-dose, respectively. There was only one sample that had detectable drug levels in each of the po groups (10 and 100 mg/kg) for LJ-001. Plasma concentrations of other compounds were greater than the lower limit of quantitation (LLOQ, 5 ng/ml) at most collection times except at the 24 hr collection for JL109 (10 mg/kg, po) and JL122 (10 mg/kg, po).

JL102 plasma levels after iv administration (10 mg/kg) showed log-linear decay up to 6 hr post-dose but thereafter showed a minimal decline up to 24 hr. JL102 was absorbed faster and was present in the plasma for longer in the ip group (10 mg/kg) than the po groups (10 and 100 mg/kg). JL102 plasma levels were higher in the 10 mg/kg po group than the 100 mg/kg po group. JL103 plasma levels after iv administration (10 mg/kg) showed log-linear decay up to 8 hr post-dose but thereafter showed minimal decline up to 24 hr. JL103 was absorbed faster and was present in the plasma for longer after ip administration (10 mg/kg) than po administration (10 and 100 mg/kg). Plasma levels were higher in the JL103 10 mg/kg po group than the 100 mg/kg group.

LJ001 (10 mg/kg iv, 5 mg/kg ip, 10 and 100 mg/kg po) showed minimal exposure due in part to a very short half-life (0.2 hr) and fast elimination (~42000 ml/hr/kg). Oral bioavailability was not determined as there was only one plasma data point in each po dose group. JL102 (10 mg/kg iv, 10 mg/kg ip, 10 and 100 mg/kg po) and JL103 (10 mg/kg iv, 10 mg/kg ip, 10 and 100 mg/kg po) had comparable plasma time courses but the PK parameters showed differences specific to dose route and level. The 10 mg/kg po groups for JL102 and JL103 showed unexpectedly lower exposure than the 100 mg/kg po groups, possibly due to significant precipitation in the GI tract for the high dose levels. The ip bioavailability for JL102 and JL103 was near 50% but the po bioavailability ranged from 3.1 to 104.7%. The three drugs distributed well into tissues.

JL109, JL118 and JL122 were rapidly absorbed after both routes with Tmax values ranging from 0.25 to 1 hr. The plasma exposure varied with the dose level and route for each compound. The plasma exposure of JL122 was relatively more dose dependent after ip and po administration in mice when compared with JL109 and JL118. Cmax measurements were 88.2, 127, and 78.2 ng/ml for JL109 and 253, 122, and 124 ng/ml for JL122 at po dose levels of 10, 50 and 100 mg/kg, respectively. Corresponding AUClast estimates were 353.91, 595.23, and 537.87 hr*ng/ml for JL-109 and 1347.67, 841.24, and 1159.83 hr*ng/ml for JL-118. The availability of JL122 in plasma increased with ip and po dose increments although not in a linear fashion. Cmax values of JL122 were 122 and 132 ng/ml at ip doses of 5 and 10 mg/kg, and 99.3 and 287 ng/ml at po doses of 10 and 100 mg/kg, respectively. Corresponding AUClast estimates were 568.61 and 924.18 hr*ng/ml at ip doses of 5 and 10 mg/kg, and 231.79 and 1271.74 hr*ng/ml at po doses of 10 and 100 mg/kg. Terminal t1/2 could not be estimated in several animals due to an insufficient number of time points with measurable concentrations in the elimination phase. MRTlast estimates were in the range of 4.5 to 10 hr (JL109), 5.3 to 11.4 hr (JL118), and 4.7 to 7.0 hr (JL122) for ip and po administration.

**CONCLUSIONS**

The PK of LJ-001 could not be compared with JL-102 and JL-103 because of the minimal plasma data available. The low exposure and very short t1/2 of LJ-001 suggests it was rapidly eliminated from the plasma and/or metabolized. LJ-001 may also have poor oral bioavailability that further exacerbated the lack of plasma data after po administration. JL-102 and JL-103 had comparable plasma time courses but the PK parameters showed differences specific to dose route and level. Chemical and/or physical properties of JL-102 and JL-103 possibly caused the disparity between oral dose level and drug exposure. The drug concentration in the GI tract for the 100 mg/kg dose level, for example, was probably well above the solubility limit and significant drug precipitation may have occurred, resulting in poorer absorption at higher doses. All drugs distributed well into tissues based on V. The plasma exposure of JL-122 was relatively more dose dependent after ip and po administration in mice when compared with the other two test compounds, JL-109 and JL-118.

**Sponsor:** National Institutes of Health**.** National Institute of Allergy and Infectious Diseases**.** Division of Microbiology and Infectious Diseases**.** Office of Biodefense Research Affairs**.** 6610 Rockledge Dr., Room 3701**.** Bethesda, MD 20892-6604

**Sponsor's Representative:** Beth Spinelli, MS, Project Officer

**NIAID DMID Contract Number: N01-AI-60011, WA#73**

**SUPPLEMENTARY REFERENCES**

1. Mayer LD, Hope MJ, Cullis PR (1986) Vesicles of variable sizes produced by a rapid extrusion procedure. Biochim Biophys Acta 858: 161-168.

2. Szoka F, Olson F, Heath T, Vail W, Mayhew E, et al. (1980) Preparation of unilamellar liposomes of intermediate size (0.1-0.2 mumol) by a combination of reverse phase evaporation and extrusion through polycarbonate membranes. Biochim Biophys Acta 601: 559-571.

3. Shui G, Stebbins JW, Lam BD, Cheong WF, Lam SM, et al. (2011) Comparative plasma lipidome between human and cynomolgus monkey: are plasma polar lipids good biomarkers for diabetic monkeys? PLoS One 6: e19731.

4. Davis B, Koster G, Douet LJ, Scigelova M, Woffendin G, et al. (2008) Electrospray ionization mass spectrometry identifies substrates and products of lipoprotein-associated phospholipase A2 in oxidized human low density lipoprotein. J Biol Chem 283: 6428-6437.
